# Hypothalamic and brainstem glucose-dependent insulinotropic polypeptide receptor neurons employ distinct mechanisms to affect feeding

**DOI:** 10.1172/jci.insight.164921

**Published:** 2023-05-22

**Authors:** Alice Adriaenssens, Johannes Broichhagen, Anne de Bray, Julia Ast, Annie Hasib, Ben Jones, Alejandra Tomas, Natalie Figueredo Burgos, Orla Woodward, Jo Lewis, Elisabeth O’Flaherty, Kimberley El, Canqi Cui, Norio Harada, Nobuya Inagaki, Jonathan Campbell, Daniel Brierley, David J. Hodson, Ricardo Samms, Fiona Gribble, Frank Reimann

**Affiliations:** 1 Institute of Metabolic Science & MRC Metabolic Diseases Unit, University of Cambridge, Cambridge, United Kingdom.; 2 Department of Neuroscience, Physiology, and Pharmacology, University College London, London, United Kingdom.; 3 Leibniz-Forschungsinstitut für Molekulare Pharmakologie (FMP), Berlin, Germany.; 4 Oxford Center for Diabetes, Endocrinology and Metabolism (OCDEM), NIHR Oxford Biomedical Research Center, Churchill Hospital, Radcliffe Department of Medicine, University of Oxford, Oxford, United Kingdom.; 5 Institute of Metabolism and Systems Research (IMSR) and Center of Membrane Proteins and Receptors (COMPARE), University of Birmingham, Birmingham, United Kingdom.; 6 Department of Metabolism, Digestion and Reproduction, Imperial College London, London, United Kingdom.; 7 Department of Medicine, Duke University Hospital, Durham, North Carolina, USA.; 8 Department of Diabetes, Endocrinology and Nutrition, Kyoto University, Kyoto, Japan.; 9 Lilly Research Laboratories, Eli Lilly and Company, Indianapolis, Indiana, USA.

**Keywords:** Metabolism, Neuroscience, Diabetes, Neuroendocrine regulation, Obesity

## Abstract

Central glucose-dependent insulinotropic polypeptide (GIP) receptor (GIPR) signaling is critical in GIP-based therapeutics’ ability to lower body weight, but pathways leveraged by GIPR pharmacology in the brain remain incompletely understood. We explored the role of *Gipr* neurons in the hypothalamus and dorsal vagal complex (DVC) — brain regions critical to the control of energy balance. Hypothalamic *Gipr* expression was not necessary for the synergistic effect of GIPR/GLP-1R coagonism on body weight. While chemogenetic stimulation of both hypothalamic and DVC *Gipr* neurons suppressed food intake, activation of DVC *Gipr* neurons reduced ambulatory activity and induced conditioned taste avoidance, while there was no effect of a short-acting GIPR agonist (GIPRA). Within the DVC, *Gipr* neurons of the nucleus tractus solitarius (NTS), but not the area postrema (AP), projected to distal brain regions and were transcriptomically distinct. Peripherally dosed fluorescent GIPRAs revealed that access was restricted to circumventricular organs in the CNS. These data demonstrate that *Gipr* neurons in the hypothalamus, AP, and NTS differ in their connectivity, transcriptomic profile, peripheral accessibility, and appetite-controlling mechanisms. These results highlight the heterogeneity of the central GIPR signaling axis and suggest that studies into the effects of GIP pharmacology on feeding behavior should consider the interplay of multiple regulatory pathways.

## Introduction

Glucose-dependent insulinotropic polypeptide (GIP) is a gut hormone released from enteroendocrine cells lining the proximal small intestine following the ingestion of a meal. GIP is a critical component of the incretin axis and, together with glucagon-like peptide-1 (GLP-1), augments postprandial insulin release through direct and indirect engagement of pancreatic β cells (1). Recent advances in pharmacology have highlighted the additional therapeutic benefits of leveraging the extrapancreatic effects of GIP signaling for the treatment of obesity.

While agonists for the GIP receptor (GIPR) given in isolation elicit modest reductions in body weight (2, 3), in preclinical and clinical studies, the GIPR signaling axis has proven to be an effective cotarget when combined with other anorexic hormones for the enhancement of weight loss, improvement of glycemic control, and the reduction of emesis (2, 4–11). For GIPR/GLP-1 receptor (GLP1R) coagonism, the resultant potent weight loss correlates with decreased food intake (12, 13), suggesting underlying central mechanisms. Indeed CNS expression of *Gipr* is necessary for the synergistic weight loss elicited by GIPR/GLP1R coagonism, indicating the importance of the GIPR signaling axis in the brain and the need to understand its role in regulating energy balance (14).

Transcriptomic analyses, FISH studies and transgenic labeling have demonstrated that key populations of brain cells are equipped to sense circulating GIP and GIP-based pharmacology directly. In rodents, cynomolgus monkeys, and humans, these *Gipr*-expressing populations localize to regions of the CNS that control eating, including the paraventricular, arcuate, and dorsomedial nuclei of the hypothalamus (PVH, ARH, and DMH, respectively) and the area postrema (AP) and nucleus tractus solitarius (NTS) of the dorsal vagal complex (DVC) (15–19). The relative contributions of *Gipr* cells within these brain regions to the control of feeding behavior are incompletely characterized.

Previously, we reported that chemogenetic activation of *Gipr* neurons in the hypothalamus acutely reduces food intake (15). Here we demonstrate that hypothalamic KO of *Gipr* failed to ablate weight loss in response to cotreatment with long-acting GIP and GLP1R agonists, suggesting that other *Gipr* neuronal populations may have an important role in regulating energy balance. Using an integrated approach incorporating chemogenetic-assisted in vivo phenotyping, circuit mapping, single-cell transcriptomics, and potentially novel fluorescently labeled GIPR agonists (GIPRAs) to identify brain regions accessible to peripherally dosed GIPRAs, we provide an in-depth characterization of *Gipr* populations in the hypothalamus, NTS, and AP. Our data support a multicenter model for central *Gipr* circuitry, where independent pathways for the control of energy balance and feeding behavior are employed by different *Gipr* populations depending on their neuroanatomical location and accessibility to GIP-based pharmacology.

## Results

**Figure 1:**
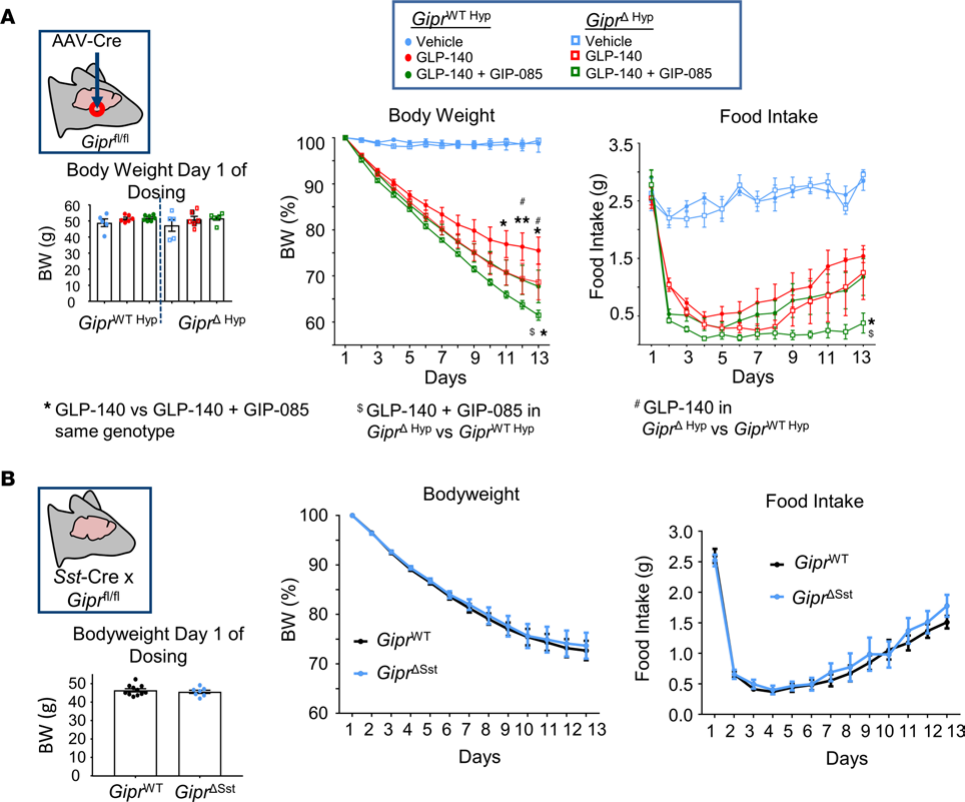
Hypothalamic *Gipr* expression is not necessary for GIPR/GLP-1R dual agonism–mediated weight loss. (**A**) DIO *Gipr*^ΔHyp^ and *Gipr*^WT^ ^Hyp^ mice were dosed with vehicle, GLP-140 (30 nmol/kg, s.c.), or GLP-140 (30 nmol/kg, s.c.) + GIP-085 (300 nmol/kg s.c.) for 12 days. (**B**) *Gipr*^ΔSst^ and *Gipr^WT^* mice were dosed with GLP-140 (30 nmol/kg, s.c.) + GIP-085 (300 nmol/kg s.c.) for 12 days. Daily body weight and food intake were measured throughout the study. Changes in body weight were calculated as a percentage of the body weight of the same animal prior to the first injection. Statistical comparisons made using a repeated measures 2-way ANOVA with a Sidak’s post hoc test. *P <0.05 GLP-140 versus GLP-140 + GIP-085 same genotype, ^$^*P* <0.05 GLP-140 + GIP-085 in *Gipr*^ΔHyp^ versus *Gipr*^WT^ ^Hyp^, ^#^*P* <0.05 GLP-140 in *Gipr*^ΔHyp^ versus *Gipr*^WT^ ^Hyp^; *n* = 5–11.

Previously, we have shown that chemogenetic activation of hypothalamic *Gipr* neurons acutely reduces food intake in mice (15). Supporting a role for the hypothalamus in mediating the effects of GIPR agonism, Zhang et al. demonstrated that peripheral administration of a GIPRA induced c-FOS in the ARH (14). To investigate whether hypothalamic *Gipr* expression is necessary for the additional weight loss elicited by GIPR/GLP1R coagonism in comparison with GLP1R agonist (GLP1RA) treatment alone, we created hypothalamic *Gipr*-KO mice (*Gipr*^Δ^ ^Hyp^) by stereotaxically injecting rAAV-Cre into the hypothalamus of *Gipr*^fl/fl^ mice or WT littermate controls (20) (Supplemental Figure 1; supplemental material available online with this article; https://doi.org/10.1172/jci.insight.164921DS1). Diet-induced obese (DIO) WT controls and *Gipr*^Δ^ ^Hyp^ mice both lost weight when treated with the long-acting GLP1RA, GLP-140 (21), alone (Figure 1A). Although *Gipr*^Δ^ ^Hyp^ mice tended to lose more weight compared with WT controls when treated with GLP-140 alone, they responded to the addition of long-acting GIPRA, GIP-085 (16), with further reduced food intake and weight loss (Figure 1A). While we cannot exclude the possibility that we have not achieved complete knockdown of *Gipr* in the *Gipr*^Δ^ ^Hyp^ model, our data suggest that fully intact hypothalamic *Gipr* expression is not required for the synergistic weight loss and anorectic activity induced by GIPR/GLP1R coagonism. Since we previously reported a high expression of somatostatin in hypothalamic *Gipr* neurons (15), we also crossed *Gipr*^fl/fl^ mice with Sst-Cre mice (*Gipr*^ΔSst^) to create mice lacking *Gipr* in *Sst*-expressing neurons. Mirroring the effects of AAV-Cre–mediated *Gipr* knockdown in the hypothalamus, responsiveness to GIPR/GLP1R agonism was preserved between WT and *Gipr*^ΔSst^ mice (Figure 1B).

**Figure 2:**
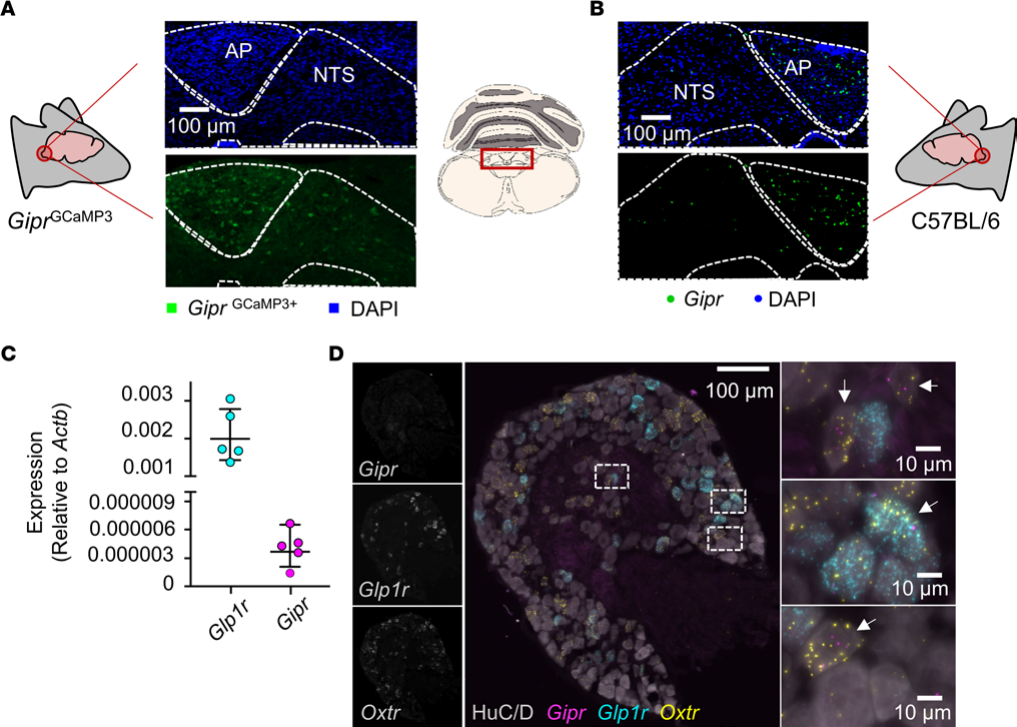
*Gipr* expression in the DVC and vagal afferents. (**A**) Coronal sections from *Gipr*^GCaMP3^ mice were stained for GFP (green). Nuclei were counterstained with DAPI (blue). Photomicrograph is representative of experiments conducted in tissue from 5 separate mice. Original magnification: ×20 (**A** and **B**). (**B**) Coronal sections of mouse brain stem tissue from C57BL/6 mice were probed for *Gipr* expression using FISH (green). Nuclei are counterstained with DAPI (blue). Photomicrograph is representative of experiments conducted in tissue from 3 separate mice. (**C**) qPCR was performed in pooled samples of nodose ganglia (*n* = 5–6 mice per replicate) for *Gipr* and *Glp1r* expression. Expression levels were calculated relative to *Actb*. Data presented as 2^ΔCT^ ± SEM. (**D**) Sections of nodose ganglia isolated from C57BL/6 mice were probed for *Gipr* (magenta), *Oxtr* (yellow), and *Glp1r* (cyan) expression using FISH. Neurons were stained for HuC/D (gray). Photomicrograph is representative of experiments conducted in tissue from 4 separate mice. Scale bars: 100 μm and 10 μm (insets). Arrows indicate *Gipr* postitive cells.

We therefore hypothesized that brain regions other than the hypothalamus could be important for the pharmacological effects of GIPR agonism. The DVC is a brain center critical for controlling food intake and is a key site of gut peptide action and receptor expression (18). Staining for GCaMP3 in serially sectioned brain tissue from *Gipr*^GCaMP3^ mice identified *Gipr*-expressing cells in the DVC of the hindbrain, specifically within the AP and the NTS (15) (Figure 2A). Using FISH, *Gipr* expression in the AP and NTS was confirmed in WT tissue, where the AP demonstrated dense *Gipr* probe localization (Figure 2B). Overlap of *Gipr* and *iCre* expression in the AP and NTS was confirmed using FISH in tissue isolated from *Gipr*-Cre mice (Supplemental Figure 2). Since vagal afferents offer an important line of communication between gut hormones and the brain, we also investigated whether *Gipr* is expressed in nodose ganglia. Both quantitative PCR (qPCR) (Figure 2C) and FISH (Figure 2D) analysis revealed that *Gipr* expression levels in nodose ganglia were on the limit of detection. We therefore focused on *Gipr* neurons in the DVC as potential modulators of feeding.

**Figure 3:**
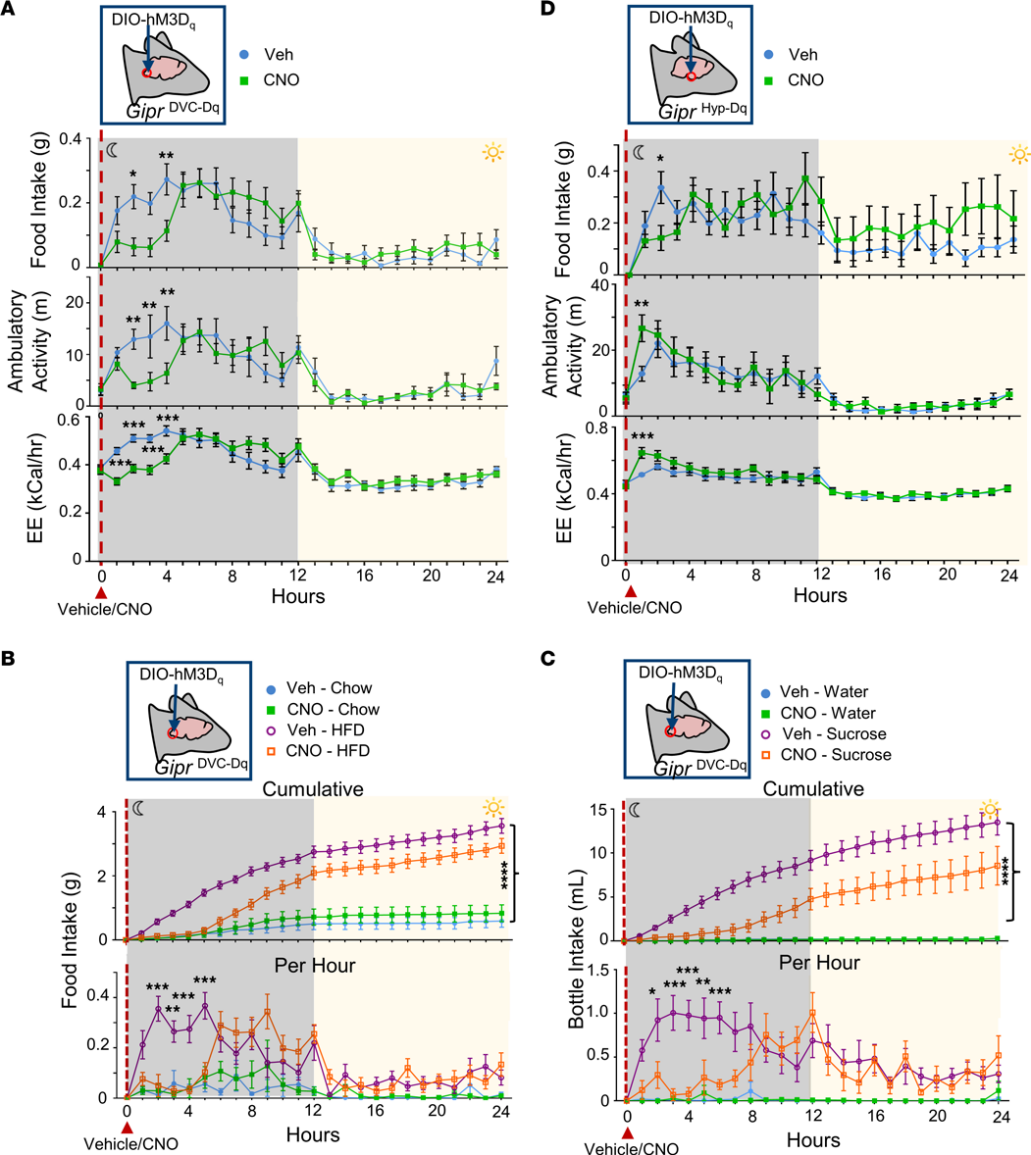
Acute activation of *Gipr* neurons in the DVC and hypothalamus reduce food intake with differing effects on energy expenditure and locomotion. (**A**–**D**) *Gipr*-Cre mice were injected with AAV-*hSyn*-DIO-hM3D(Gq)-mCherry into the DVC (**A**–**C**) or the hypothalamus (**D**) to produce *Gipr*^DVC–Dq^ or *Gipr*^Hyp–Dq^ mice, respectively. Mice were housed in indirect calorimetry cages equipped with continuous monitoring. CNO (1 mg/kg) or vehicle was injected i.p. at the onset of the dark phase. Mice were given standard chow and drinking water (**A** and **B**), a choice of standard chow or 45% HFD and drinking water (**C**), or standard chow with a choice of drinking water or 10% sucrose (**D**). Data are plotted as mean ± SEM. Statistical comparisons made using a repeated measures 2-way ANOVA with a Sidak’s post hoc test. *P < 0.05, **P < 0.01, ***P < 0.001, ****P < 0.0001; *n* = 8–16.

To investigate whether *Gipr* neurons in the DVC are involved in regulating energy balance, we used chemogenetics to acutely manipulate their activity. *Gipr*-Cre mice were injected with an rAAV carrying the Cre-inducible G_q_-coupled designer receptor exclusively activated by designer drugs (DREADD), hM3Dq (rAAV-*hSyn*-DIO-hM3D[Gq]-mCherry) (22, 23), designed to preferentially target neurons (24, 25), into the DVC to produce *Gipr*^DVC–Dq^ mice. Using a crossover design study, the metabolic effects of activating hM3Dq receptors with clozapine-*N*-oxide (CNO) in *Gipr*^DVC–Dq^ mice were monitored continuously in an indirect calorimeter (Supplemental Figure 3A). In chow-fed *Gipr*^DVC–Dq^ mice, the acute activation of *Gipr* neurons in the DVC suppressed food intake, ambulatory activity, and energy expenditure (Figure 3A). Concomitant water intake and respiratory exchange ratio (RER) were also significantly reduced (Supplemental Figure 3B). The ability of acute activation of *Gipr*^DVC–Dq^ neurons to suppress feeding and fluid intake was irrespective of substrate palatability (Figure 3, B and C). Parallel experiments with hM3Dq targeted to the hypothalamus (*Gipr*^Hyp–Dq^) recapitulated our previous published inhibition of food intake (15); however, in contrast to *Gipr*^DVC–Dq^ mice, *Gipr*^Hyp–Dq^ mice showed increased ambulatory activity and energy expenditure after CNO injection (Figure 3D), suggesting that *Gipr* neurons in both the hypothalamus and the hindbrain contribute to the control of feeding behavior and the regulation of energy homeostasis but signal through the recruitment of different circuitry.

**Figure 4:**
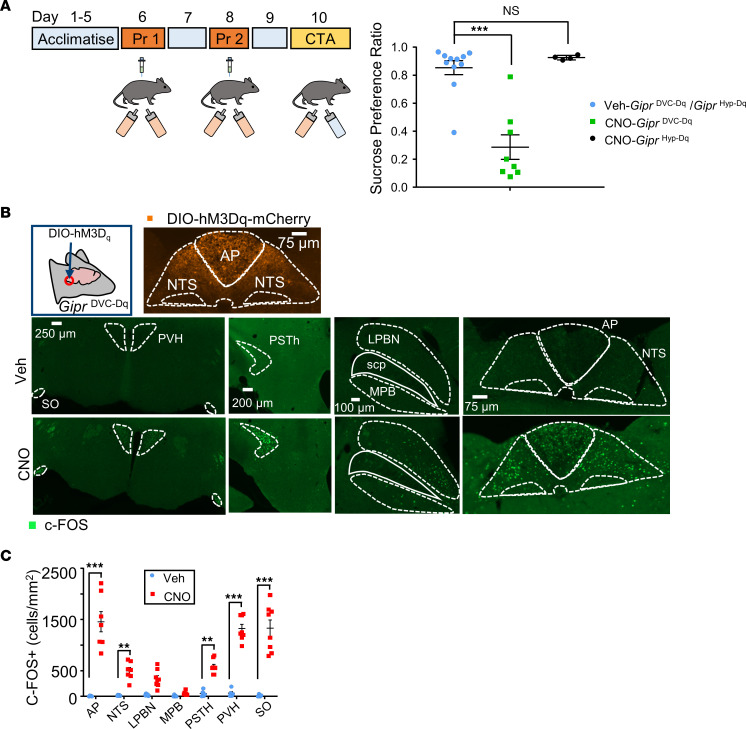
Acute activation of *Gipr* neurons in the DVC induces conditioned taste avoidance. (**A**) Schematic illustrating CTA protocol. Taste avoidance conditioned by CNO (1 mg/kg) or vehicle administration in *Gipr*^DVC–Dq^ and *Gipr*^Hyp–Dq^ mice. Data are plotted as mean ± SEM. Statistical analysis performed using a 1-way ANOVA with a Dunnett’s post hoc test. ***P < 0.001; *n* = 4–11. (**B**) c-Fos activation (green) in the DVC from *Gipr*^DVC–Dq^ mice following administration of CNO or vehicle. Image of hM3Dq-mCherry staining in DVC from *Gipr*^DVC–Dq^ mouse representative of *n* = 13 experiments. Images of c-Fos staining are representative of *n* = 5–8 experiments. (**C**) Quantification of c-Fos in indicated brain regions. Data are plotted as mean ± SEM. Statistical comparisons made using a repeated measures 2-way ANOVA with a Sidak’s post hoc test. **P < 0.01, ***P < 0.001; *n* = 5–8. PVH, paraventricular nucleus of the hypothalamus; SO, supraoptic nucleus; PSTh, parasubthalamic nucleus; LPBN, lateral parabrachial nucleus; MPBN, medial parabrachial nucleus; scp, superior cerebellar peduncle; AP, area postrema; NTS, nucleus tractus solitarius.

Inhibition of food and liquid intake and ambulatory activity suggest that the acute chemogenetic activation of populations of *Gipr* neurons in the hindbrain could induce malaise. To investigate whether hindbrain *Gipr* neurons may engage neural circuitry underlying nausea and avoidance, we performed conditioned taste avoidance (CTA) assays as previously described (26). The average preference ratio for 5% sucrose versus plain water was reduced following pairing of the sucrose with acute CNO-mediated activation of *Gipr*^DVC–Dq^ neurons compared with vehicle-treated *Gipr*^DVC–Dq^ control mice. In contrast, CNO-mediated activation of *Gipr*^Hyp–Dq^ neurons had no effect on preference for 5% sucrose when compared with vehicle-treated *Gipr*^Hyp–Dq^ mice (Figure 4A).

To establish which brain regions are recruited following *Gipr*^DVC–Dq^ activation, c-FOS mapping was performed. In CNO-treated *Gipr*^DVC–Dq^ mice, c-FOS labeling was increased in both the AP and the NTS compared with vehicle-treated *Gipr*^DVC–Dq^ mice, demonstrating local neuronal activation following hM3D engagement (Figure 4B). c-FOS was also significantly increased in CNO-treated *Gipr*^DVC–Dq^ mice in the parasubthalamic nucleus (PSTh), paraventricular nucleus of the hypothalamus (PVH), the supraoptic nucleus (SO), and — while not significant — trended toward increase in the lateral parabrachial nucleus (LPBN) (Figure 4C). These data indicate that chemogenetic activation of *Gipr* neurons in the hindbrain recruits nuclei crucial for energy balance and meal termination in distant brain regions, in addition to circuits local to the hindbrain.

**Figure 5:**
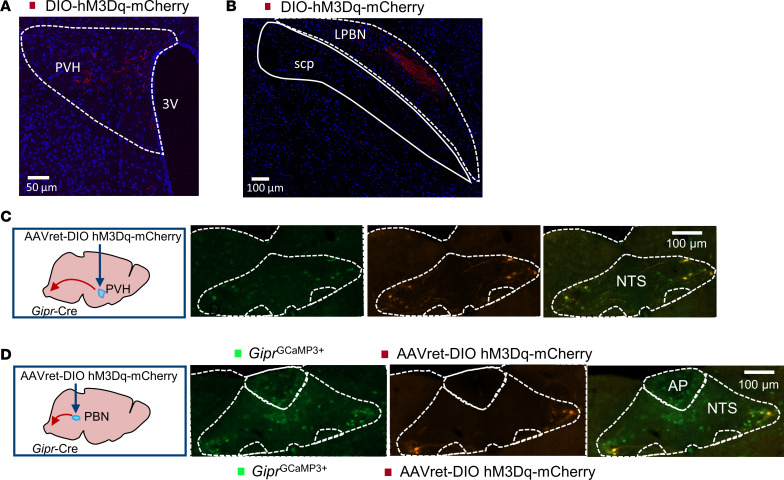
*Gipr* neurons in the NTS project to the PBN and PVH. (**A** and **B**) *Gipr*-Cre mice were injected with AAV-*hSyn*-DIO-hM3D(Gq)-mCherry into the DVC. mCherry^+^ fibers were visualized by IHC in serial brain sections localising in the PVH (**A**) and LPBN (**B**) Scale bars: 50 μm (**A**), 100 μm (**B**). (**C** and **D**) AAVs optimized for retrograde transport packaging *hSyn*-DIO-hM3D(Gq)-mCherry (AAVret-DIO hM3Dq-mCherry) were injected into the PVH (**C**) and the LPBN (**D**). mCherry^+^ cell bodies were visualized in the DVC by IHC. Photomicrographs are representative of experiments conducted in tissue from 3 separate mice. PVH, paraventricular nucleus of the hypothalamus; LPBN, lateral parabrachial nucleus. Scale bars: 100 μm.

Recruitment of brain regions as measured by c-FOS mapping could indicate either direct or secondary activation. The *hSyn*-DIO-hM3D(Gq)-mCherry construct injected to create *Gipr*^DVC–Dq^ mice encoded a mCherry fluorescent tag fused to the hM3Dq receptor that was expressed in the axonal processes and the soma of targeted cells. To determine whether *Gipr* neurons of the DVC project to and potentially directly activate regions with increased c-FOS labeling, serial sections from *Gipr*^DVC–Dq^ mice were stained for mCherry fluorescent fibers. mCherry^+^ projections were clearly identified in the PVH (Figure 5A) as well as the LPBN (Figure 5B).

To further delineate which DVC *Gipr* neurons project to brain regions associated with meal termination and nausea, rAAV with enhanced retrograde uptake packaging the *hSyn*-DIO-hM3D(Gq)-mCherry construct was delivered to the PVH and the LPBN (27). Cell bodies expressing hM3D(Gq)-mCherry were located in the caudal NTS (Figure 5, C and D) but not the AP, consistent with previous observations that *Gipr* neurons within the AP have limited projections outside the DVC (28).

**Figure 6:**
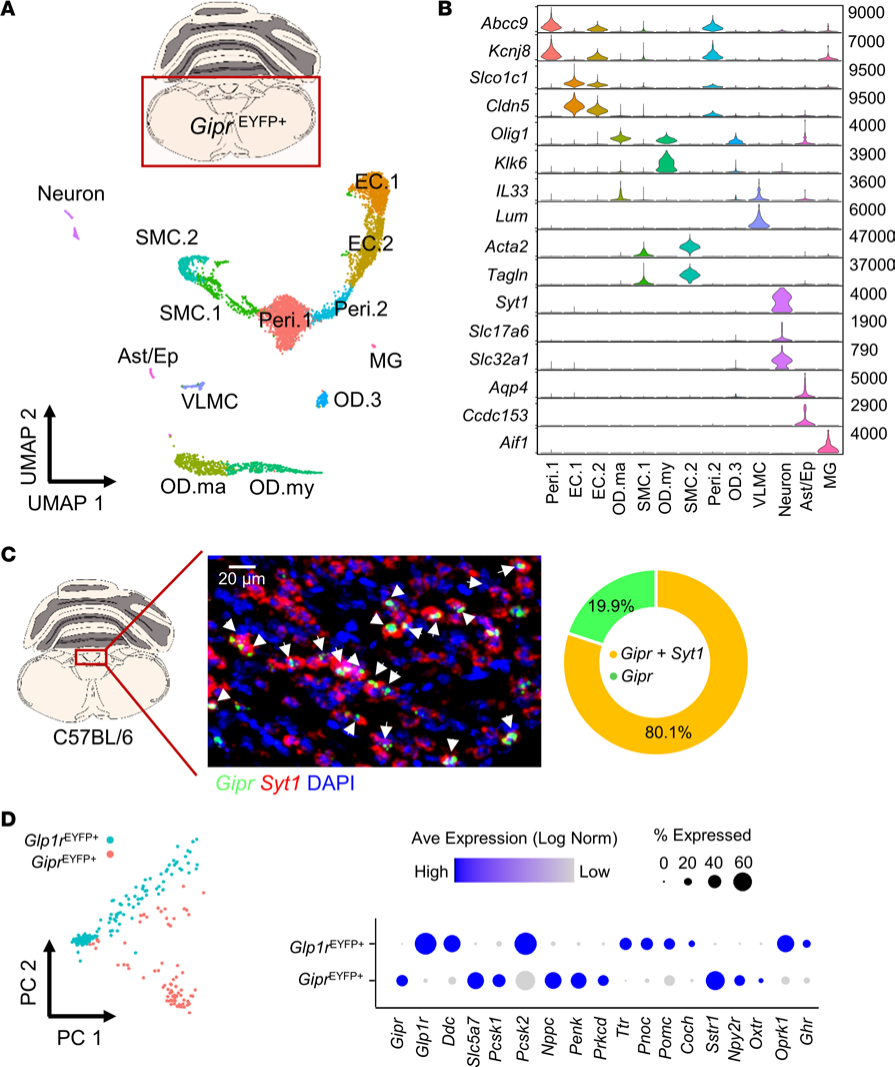
Transcriptomic characterization of *Gipr*-expressing cells in the hindbrain. *Gipr* cells were isolated from single-cell digests of hindbrain sections from *Gipr*^EYFP^ mice via FACS, and their transcriptomes were characterized via scRNA-Seq followed by clustering analysis. (**A**) Uniform Manifold Approximation and Projection (UMAP) visualization of *Gipr*^EYFP+^ cells. Cell types were assigned according to expression of marker genes (Peri.1, Peri.2 = pericytes; EC.1, EC.2 = endothelial cells; OD.ma = mature ODs; OD.my = myelinating ODs; OD.3 = ODs, SMC.1, SMC.2 = smooth muscle cells; VLMC = vascular leptomenigeal cells; Neuron = neurons; Ast/Ep = astroependymal cells; MG = microglia) (**B**). (**C**) Dual-label FISH showing colocalization of *Gipr* (green) and *Syt1* (red) transcript in coronal sections of mouse brain stem tissue from C57BL/6 mice. Nuclei are counterstained with DAPI (blue). *Gipr*/*Syt1* coexpression was quantified in sections from 3 mice. Scale bar: 20 μm. Arrows indicate cells expressing both *Gipr* and *Syt1*. (**D**) Principal component analysis of *Gipr*^EYFP+^ versus *Glp1r*^EYFP+^ neurons (left). Dot plot of selected differentially expressed genes in *Gipr*^EYFP+^ versus *Glp1r*^EYFP+^ neurons (right).

To better understand the cell types expressing *Gipr* in the DVC, we created a transcriptomic profile of *Gipr* cells isolated from the hindbrain. *Gipr*-Cre mice were crossed with an EYFP reporter strain to produce *Gipr*^EYFP^ mice as previously described (15). FACS-purified *Gipr*^EYFP+^ cells were collected from cell suspensions prepared from sections of medulla oblongata containing the DVC from *Gipr*^EYFP^ mice. The transcriptomes of captured *Gipr*^EYFP+^ cells were analyzed via single-cell RNA-Seq (scRNA-Seq), yielding a data set encompassing 5,521 cells. Unsupervised clustering analysis revealed vast diversity in *Gipr*^EYFP+^ cells of the hindbrain, with *Gipr*^EYFP+^ cells clustering into 13 separate subpopulations. Cell type identities were assigned based on the expression of canonical marker genes (29–31), identifying clusters of neurons (*Syt1*, *Slc17a6*, *Slc32a1*), oligodendrocytes (ODs) (*Olig1*), mature ODs (*Il33*), myelinating ODs (*Klk6*), astroependymal cells (*Aqp4*, *Ccdc153*), microglia (*Aif1*), pericytes (*Abcc9*, *Kcnj8*), vascular smooth muscle cells (VSMC) (*Acta2*, *Tagln*), endothelial cells (ECs) (*Slco1c1*, *Cldn5*), and vascular and leptomeningeal cells (VLMC) (*Lum*) (Figure 6, A and B). FISH analysis in brains from WT mice demonstrated that, within the DVC, the majority of *Gipr*-expressing cells coexpressed the neuronal marker *Syt1* (Figure 6C), and further transcriptomic analysis focused on the *Gipr*^EYFP+^ neuronal population.

To investigate *Gipr*^EYFP+^ neurons further, cells were filtered for expression of the neuronal markers *Syt1* and *Snap25*. Contaminating ODs, pericytes, and astroependymal cells were excluded based on expression of *Olig1*, *Abcc9*, and *Aqp4*, respectively, resulting in 79 *Gipr*^EYFP+^ neurons. We compared the transcriptomes of *Gipr*^EYFP+^ neurons with 199 hindbrain *Glp1r*^EYFP+^ neurons isolated from *Glp1r*^EYFP^ mice (Supplemental Figure 4). As previously reported (32–34), *Gipr*^EYFP+^ and *Glp1r*^EYFP+^ populations of the hindbrain are largely separate and distinct. *Gipr*^EYFP+^ neurons were enriched for transcripts encoding the neuropeptides natriuretic peptide C (*Nppc*) and proenkephalin (*Penk*), and protein kinase C δ (*Prkcd*). *Glp1r*^EYFP+^ neurons were enriched for the neuropeptides prepronociceptin (*Pnoc*) and proopiomelanocortin (*Pomc*) as well as the thyroid hormone transporter transthyretin (*Ttr*) (Figure 6D). A small population of *Gipr/Glp1r* coexpressing neurons were observed in the hindbrain and localized to the spinal trigeminal nucleus (Sp5I) (Supplemental Figure 5).

**Figure 7:**
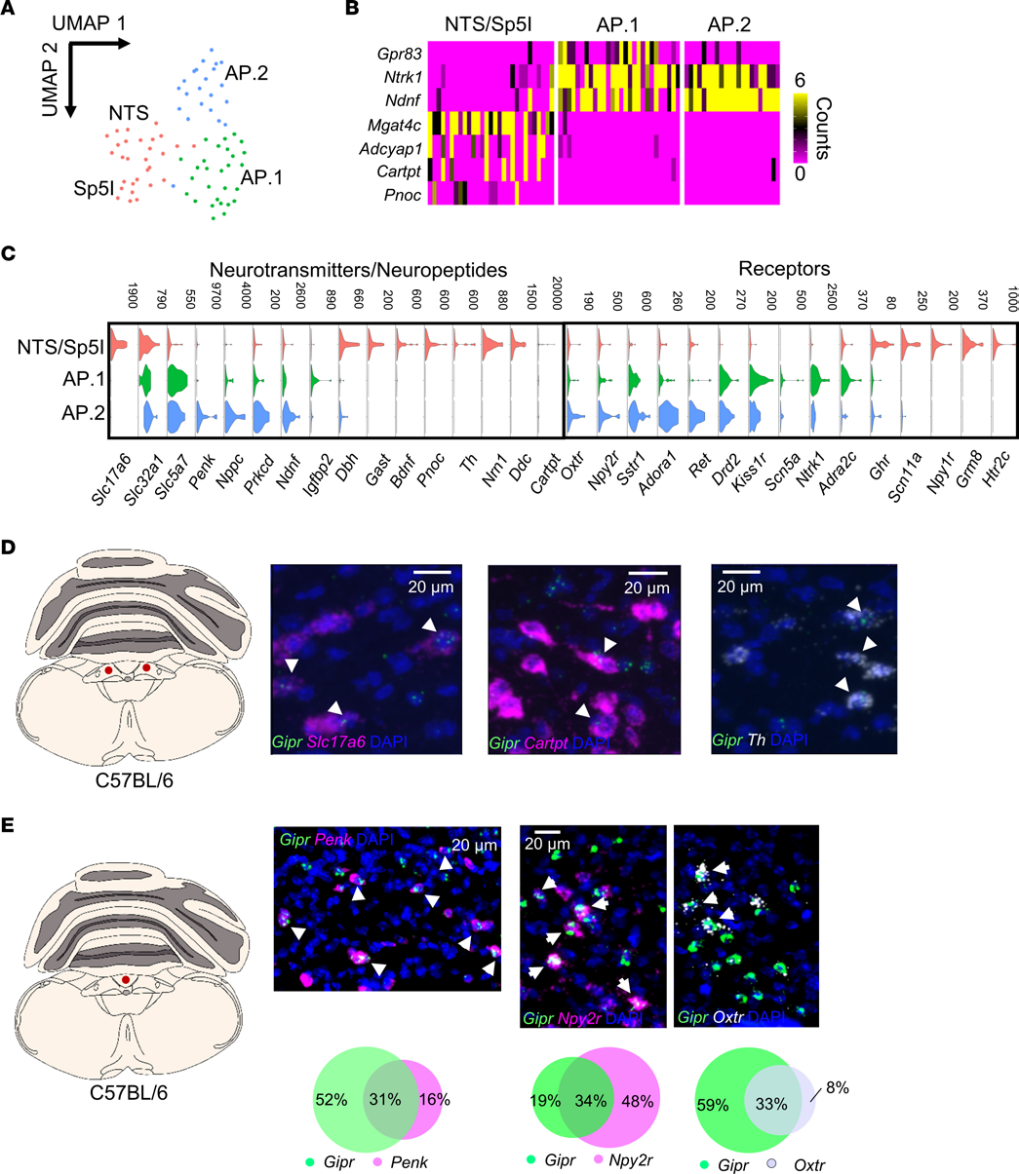
Transcriptomic analysis of *Gipr* Neurons in the hindbrain. (**A**) Uniform Manifold Approximation and Projection (UMAP) showing clusters of isolated *Gipr* neurons following dimensionality reduction and unsupervised clustering. (**B**) The top 15 markers for each cluster were cross-referenced with published brain region–specific transcriptional markers and the Allen Brain Atlas for region specific cluster assignments. (**C**) Violin plots of neurotransmitters, secreted products, and cell surface receptors or ion channels enriched in each cluster. Data are plotted in CPM. (**D**) Dual-label FISH showing colocalization of *Gipr* with either *Slc17a6*, *Cartpt*, or *Th* transcript in the NTS of brain stem tissue from C57BL/6 mice. Nuclei are counterstained with DAPI (blue). (**E**) Dual-label FISH showing colocalization of *Gipr* with either *Penk*, *Npy2r*, or *Oxtr* transcript in the AP of brain stem tissue from C57BL/6 mice. Nuclei are counterstained with DAPI (blue). *Gipr*/*Syt1*, *Gipr*/*Npy2r*, or *Gipr*/*Oxtr* coexpression was quantified in sections from 3 mice. Scale bars: 20 μm. Arrows represent colocalization of probes as indicated.

Unsupervised clustering revealed that *Gipr*^EYFP+^ neurons formed 3 clusters expressing markers of both glutamatergic (*Slc17a6*) and GABAergic (*Slc32a1*) cells. In keeping with a recent report cataloging AP neuron transcriptomic profiles (33, 34), the majority of *Gipr*^EYFP+^ neurons were GABAergic, with 1 of the 3 clusters also expressing *Slc17a6*. To infer the anatomical distribution of these clusters, the top 15 differentially expressed genes for each cluster were compared with available single-nucleus RNA-Seq data sets and were mapped to the Allen Brain Atlas (32, 34, 35). The 2 GABAergic clusters expressed markers indicating they originated from the AP (AP.1, AP.2), while the remaining *Slc32a1*^+^/*Slc17a6*^+^ cluster contained cells expressing NTS markers and a small subpopulation of cells expressing markers from the Sp5I (NTS/Sp5I) (Figure 7, A and B, and Supplemental Figure 6).

Given our finding that *Gipr* neurons of the NTS exhibit projection patterns that are distinct from *Gipr* neurons of the AP, we hypothesized that *Gipr*^NTS^ and *Gipr*^AP^ neurons may be separate and distinct populations that engage different signaling mechanisms. We therefore performed differential gene expression analysis to compare and contrast neuropeptides and cell-surface receptors that characterize *Gipr*^NTS^ versus *Gipr*^AP^ neurons. The NTS/Sp5I cluster was distinct from the AP clusters in its enrichment for dopamine β-hydroxylase (*Dbh*), tyrosine hydroxylase (*Th*), and dopa decarboxylase (*Ddc*). Neuropeptides enriched in the NTS/Sp5I cluster included cocaine- and amphetamine-regulated transcript protein (*Cartpt*) and gastrin (*Gast*) (Figure 7C). Coexpression of either *Th* or *Cartpt* with *Gipr* in the NTS was confirmed using FISH (Figure 7D).

In contrast, the AP clusters were enriched for *Penk* and *Nppc*. FISH revealed that the majority of *Penk* neurons in the AP coexpress *Gipr,* suggesting that GIP may play a role in regulating endogenous opioid signaling in the hindbrain (Figure 7E). Analysis of cell-surface receptor expression revealed that hindbrain *Gipr*^AP^ neurons are enriched for *Npy2r*, the receptor for peptide YY (PYY), and the oxytocin receptor (*Oxtr*). Quantitative FISH analysis demonstrated that the majority of *Gipr*^AP^ neurons coexpress *Npy2r* and that the majority of oxytocin-sensing neurons of the AP express *Gipr* (Figure 7E).

**Figure 8:**
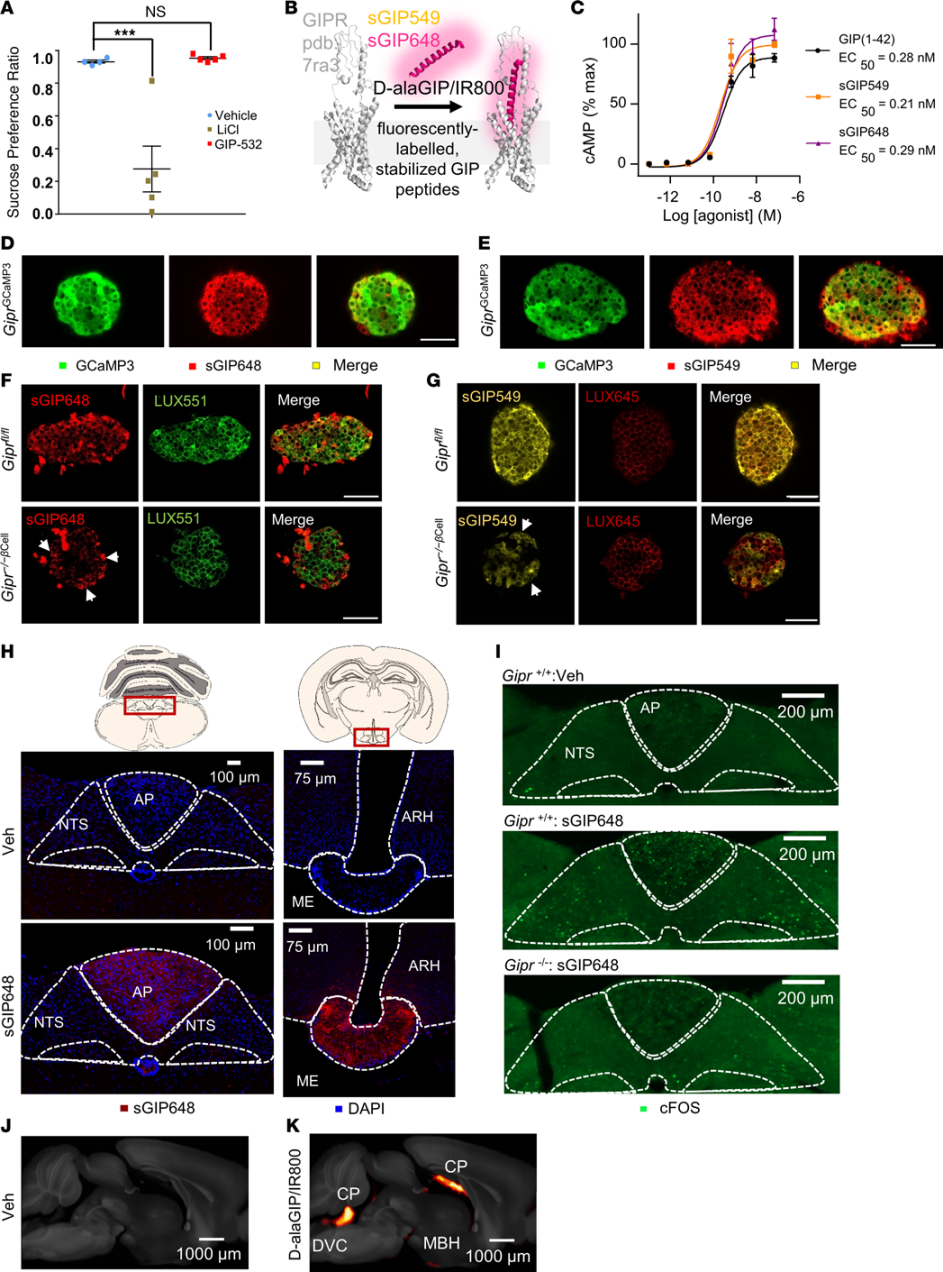
Stabilized, fluorescently labeled GIP peptides are specific and effective GIPR agonists that access circumventricular organs in the CNS. (**A**) Taste avoidance conditioned by vehicle or GIP-532 (10 nmol/kg, s.c.) or LiCl (0.4M, i.p.) in WT mice. Data are plotted as mean ± SEM. Statistical analysis performed using a 1-way ANOVA with a Dunnett’s post hoc test. *n* = 4–5. ***P < 0.001. (**B**) Schematic showing nature and binding of the stabilized red (sGIP549) and far red (sGIP648) GIPR probes (GIPR pdb: 7ra3). (**C**) sGIP549, sGIP648, and native GIP(1-42) cAMP signaling responses in T-REx-SNAP-GIPR cells, *n* = 3. (**D** and **E**) sGIP648 (**D**) and sGIP549 (**E**) label *GIPR^GCaMP3^* reporter islets, showing colocalization with GCaMP3^+^ cells (*n* = 11-12 islets, 2 animals). (**F**) sGIP648 labels β cells, identified using LUX551, in *Gipr^fl/fl^* control but not *Gipr^–/–βcell^* islets. Arrows show GIPR labeling only in LUX551^–^ cells, presumed to be α cells (*n* = 63 islets, 10 animals). (**G**) sGIP549 labels β cells, identified using LUX645, in *Gipr^fl/fl^* control but not *Gipr^–/–βcell^* islets. Arrows show GIPR labeling only in LUX645^–^ cells, presumed to be α cells (*n* = 56 islets, 10 animals). (**H**) sGIP648 labels the DVC and MBH following i.v. administration in mice. (Veh: *n* = 4, sGIP648: *n* = 7). (**I**) c-Fos activation in the DVC following i.v. injection of vehicle or sGIP648 into *Gipr*^+/+^ or *Gipr*^–/–^ mice (*n* = 3 mice per genotype per treatment). (**J** and **K**) Maximum intensity projection of the average signal computed from individual brains (*n* = 4) overlaid onto the Common Coordinate Framework V3 template from AIBS for mice treated with vehicle (**J**) or D-alaGIP/IR800 (**K**). CP, choroid plexus; DVC, dorsal vagal complex; MBH mediobasal hypothalamus; ARH, arcuate nucleus of the hypothalamus; ME, median eminence; NTS, nucleus tractus solitarius; AP, area postrema; Scale bar: 53 μm (**D**–**G**).

Having observed a strong anorexic and CTA response when targeting the DVC chemogenetically, we were intrigued by recent reports that pharmacological activation of GIPR ameliorates nausea and emesis in response to other noxious agents (2, 16, 17, 28). We, thus, tested if peripheral administration of a short-acting GIPRA, GIP-532 (2), induced CTA in WT animals. In agreement with other studies (2, 16, 17), pharmacological GIPR agonism did not evoke CTA (Figure 8A). Following our circuit tracing and expression analyses of *Gipr* DVC neurons (Figures 5–7), we hypothesized that chemogenetic activation of *Gipr* neurons in the NTS, rather than the AP, underlie the CTA response we observed in *Gipr*^DVC–Dq^ mice but that these are not the primary *Gipr* neuronal population accessed and engaged by peripherally administered GIPRAs. To test this hypothesis, we aimed to clarify CNS access of GIP-based peptide agonists. For this purpose, we designed fluorescently labeled, stabilized GIPR peptide agonist probes sGIP549 and sGIP648 (Figure 8B), which were initially validated against heterologously expressed receptors in HEK293 cells and native receptors in pancreatic islets. Both sGIP549 and sGIP648 demonstrated functional specificity for GIPR over GLP1R in HEK293 cells expressing SNAP-tagged receptors (Supplemental Figure 7, A and B). Analysis of cAMP signaling potencies in HEK293 cells overexpressing GIPR showed that both 549 and 648 conjugates retained the pharmacological characteristics of native GIP (Figure 8C). sGIP648 stimulated similar GIPR internalization to native GIP (Supplemental Figure 7C). In keeping with high expression levels of *Gipr* in pancreatic α, β, and δ cells (15, 36), treatment of whole islets isolated from *Gipr*^GCaMP3^ mice with sGIP549 or sGIP648 revealed extensive colocalization of GCaMP3 with the fluorescently labeled GIPR peptide agonists (Figure 8, D and E). Further demonstrating specificity, sGIP549 and sGIP648 were unable to label β cells conditionally deleted for *Gipr* (*Gipr*^–/–βCell^), whereas labeling with the fluorescent GLP1R antagonist, LUX645 (37), remained unchanged (Figure 8, F and G, and Supplemental Figure 7, D–F).

Having established that fluorescently labeled stabilized GIPR peptide agonists exhibit specificity and potency for GIPR, we mapped brain regions accessible to peripherally administered GIPRAs. Serial coronal sections from mice dosed acutely with i.v. administration of either vehicle or sGIP648 were imaged. sGIP648 localized to circumventricular organs (CVO) in the brain, including the AP and the median eminence (ME) in the hypothalamus, with markedly less fluorescence signal observed in the bordering ARH and NTS regions (Figure 8H). Demonstrating the specificity of sGIP648 for GIPR in vivo, peripherally administered sGIP648 failed to induce c-FOS activation in the DVC of *Gipr*-KO animals (Figure 8I). To further characterize central access of GIPRAs, we utilized 2 additional fluorescently labeled GIPRAs, D-alaGIP/IR800 and GIP-532/IR800 (Supplemental Figure 7). Whole-brain imaging was performed with light sheet fluorescence microscopy in brains harvested from mice dosed s.c. with either vehicle or D-alaGIP/IR800. Robust fluorescent labeling was observed in the choroid plexus, DVC, and mediobasal hypothalamus, with limited labeling in brain regions shielded by the blood-brain barrier (BBB) (Figure 8, J and K, and Supplemental Figure 8).

## Discussion

Though recent work has demonstrated that *Gipr* is expressed extensively throughout the CNS (15), and that central *Gipr* expression is necessary for GIPR/GLP1R coagonism to lower body weight beyond GLP1R agonism alone (14), uncertainty surrounding the brain regions and central signaling pathways mediating this effect remain. In this study, we characterized *Gipr*-expressing neurons in the hypothalamus and the DVC and interrogated their role in controlling feeding behavior, finding that *Gipr* neurons leverage different anorexigenic pathways depending on their neuroanatomical location. Regional differentiation between *Gipr* neuronal populations was also present within the DVC, where *Gipr* neurons of the AP and the NTS were distinct in their connectivity and gene expression profiles. Our data support a multicenter model for the central GIPR signaling axis, where *Gipr* populations engage independent modes of behavioral regulation in a region-specific manner to affect feeding and energy balance.

### Gipr neurons in the DVC engage separate anorexigenic pathways from Gipr neurons in the hypothalamus.

Our GIPRA labeling studies indicating that the hypothalamus and DVC are key target regions for peripherally administered GIPRAs are in accordance with previous work demonstrating increased c-FOS expression in the hypothalamus and hindbrain following GIPRA treatment (2, 14, 16, 17). Using chemogenetics, we probed the effects of acute activation of *Gipr* neuronal populations in either the hypothalamus or the DVC on feeding behavior. While stimulation of *Gipr* neurons in both regions suppressed dark-phase food intake, *Gipr*^Hyp–Dq^ activation resulted in transient increased ambulatory activity with no effect on CTA. By contrast, *Gipr*^DVC–Dq^ activation reduced ambulatory activity and energy expenditure, and triggered CTA, indicating that *Gipr*^Hyp–Dq^ and *Gipr*^DVC–Dq^ populations engage distinct anorexigenic mechanisms. While meal-patterning analysis was not conducted in this study, c-FOS mapping following *Gipr*^DVC–Dq^ activation indicated that *Gipr*^DVC^ neurons engage brain centers implicated in meal termination, including the AP, NTS, LPBN, PSTh, and the PVH, similar to other appetite-modulating hormones and pharmacological agents such as long-acting GLP1RAs, PYY and amylin, and illness-producing agents such as LiCl (2, 16, 38–41).

Recent studies have shown that GIPR agonism attenuates nausea and aversion in preclinical models. Specifically, coadministration of a GIPRA decreased emesis and kaolin intake in GLP1RA-treated musk shrews and rats (16) and reduced CTA in mice in response to a PYY analogue and GDF-15 (2, 28). GIPRA-mediated suppression of aversion is hypothesized to proceed through GABAergic *Gipr* neurons in the AP acting as local inhibitory modulators of neurons that sense circulating toxins and emetic stimuli (16). In our *Gipr*^DVC–Dq^ model, the stimulatory hM3Dq DREADD receptor was expressed in both the AP and the NTS *Gipr* populations. Given evidence supporting an antiaversive mechanism of action for *Gipr*^AP^ neurons, our data demonstrating that *Gipr*^DVC–Dq^ activation conditions taste aversion suggest that the *Gipr*^NTS^ and *Gipr*^AP^ populations have opposite effects on circuits regulating nausea.

### Gipr neurons of the AP and the NTS are differentially networked and transcriptomically distinct.

While *Gipr*^AP^ neurons form dense arborizations that are restricted to the DVC (28), viral-assisted circuit mapping revealed that *Gipr*^NTS^ neurons project to the LPBN and the PVH. The PVH represents a critical hub for the central control of energy balance, integrating a diverse range of nutritionally related hormonal and synaptic inputs, and has been previously demonstrated to receive inputs from the NTS (18, 42). Similarly, the LPBN integrates visceral and gustatory information from the brainstem together with hypothalamic inputs to regulate feeding, nausea, and blood glucose (43–46). Transcriptomically, *Gipr*^NTS^ neurons were enriched for *Th* and *Dbh* expression compared with the *Gipr*^AP^ population, suggesting that these cells are noradrenergic. *Dbh*-expressing neurons in the NTS have previously been shown to promote anorexia by directly innervating and activating CGRP^LPBN^ neurons (47). Our data demonstrating *Gipr*^DVC–Dq^ activation–mediated anorexia, combined with the transcriptomic identity and connectivity mapping of the *Gipr*^NTS^ neurons, are consistent with *Gipr*^NTS^ cells belonging to this same *Dbh*^NTS→LPBN^ circuit.

In accordance with previous reports (32–34), our transcriptomic analysis confirmed that *Gipr*^AP^ neurons predominantly expressed *Slc32a1* and are GABAergic. Indeed whole-cell patch-clamp recordings demonstrated that GIP-evoked hyperpolarization of *Gfral*^AP^ neurons is sensitive to GABA_A_ receptor blockade (28). This local inhibitory circuit is hypothesized to underlie decreased c-FOS activation in the AP upon GIPRA/GLP1RA coadministration compared with GLP1R monoagonism (16, 17). However this mechanism may not be translatable to GIPRA-dependent attenuation of PYY-mediated aversion, as GIPR coagonism increased c-FOS expression in the AP compared with PYY treatment alone (2). Similar to studies in rat and nonhuman primate tissue (2), we found that the majority of *Gipr*^AP^ neurons coexpress *Nyp2r* but do not coexpress *Glp1r*. Therefore, GIP-mediated attenuation of aversion and emesis may proceed through multiple pathways, depending on whether *Gipr* is coexpressed in the target AP neuron population of a given agent. Our finding that the majority of *Penk*-expressing cells in the AP coexpressed *Gipr* suggests that GIP-mediated endogenous opioid signaling may provide additional mechanisms underlying GIP’s regulatory effects on aversion and feeding.

### Peripherally administered GIPRAs access CVOs.

An important consideration for understanding how peripherally derived agents affect central circuits is the relative accessibility of their target cell populations within the CNS. One working model to describe the central effects of gut hormones and their pharmacological analogues postulates their passive diffusion across the BBB (48–50). However, previous studies using fluorescently labeled GLP-1, liraglutide, and semaglutide demonstrated that GLP-1 and GLP1RAs largely did not permeate the BBB and, instead, worked through select *Glp1r*-expressing regions in close proximity to the cerebral ventricles (40, 51, 52). Using specific, stabilized fluorescently labeled GIP peptide analogues, we found that peripherally administered GIPRAs principally localized to CVOs, with detectable but decreased labeling in directly apposing brain regions such as the ARH. Our data demonstrate that, similar to GLP-1–based pharmacology, the BBB restricts the direct access of GIPRAs to target cell populations located in CVO entry points.

This finding does not, however, preclude involvement of cell populations in neighboring brain regions mediating GIPRA-dependent effects on appetite. Circulating regulatory signals are thought to activate cells in brain regions proximal to CVOs through fenestrated capillaries, such as those in the median eminence-ARH barrier (48, 53). Additionally, neuronal populations protected by the BBB have been shown to send axon terminals to CVOs, suggesting that such neurons can sense regulatory agents distally (54). The unique chemical properties and design of a GIPRA, including peptide stability and albumin binding, may affect how different pharmaceuticals interact with target *Gipr* populations. Indeed, the C16 acylated GLP-1RA liraglutide shows greater access to the PVH compared with the C18 acylated GLP-1RA semaglutide (40). Therefore, the brain regions and central mechanisms underpinning the effects of individual GIPRAs may differ depending on their central availability, and *Gipr* populations residing both within and directly apposing CVOs present pharmacologically relevant targets.

### Summary.

Here we demonstrate that *Gipr* neurons in different brain regions are transcriptomically distinct and differentially networked, engage distinct pathways to modulate energy balance, and exhibit differing accessibility to peripherally administered GIPRAs. Mechanisms underlying the actions of current and future GIPR-based therapeutics could, therefore, depend on the balance of *Gipr* populations engaged across distinct neuroanatomical locations.

## Methods

Supplemental Methods are available online with this article. Raw scRNA-Seq data have been deposited into the NCBI GEO database (GSE228192).

### Statistics.

Data are presented as mean ± SEM. Statistical analysis was performed using Microsoft Excel, GraphPad Prism 7.0, and Seurat V4. For all statistical tests, an α risk of 5% was used. Multiple comparisons were made using 2-way ANOVA or a repeated-measures 2-way ANOVA. Sample size was computed based on pilot data and previously published data. n values represent the number of mice or biological replicates used in each study unless otherwise indicated in the figure legend.

### Study approval.

All animal studies were approved by the University of Cambridge and University of Birmingham Animal Welfare and Ethical Review Body and by the Duke University and Eli Lilly and Co. Institutional Animal Care and Use Program. Work conformed to the Animals (Scientific Procedures) Act 1986 Amendment Regulations (SI 2012/3039) and was performed under the UK Home Office Project Licenses PE50F6065, P2ABC3A83, and PP1778740.

## Author contributions

AA, JB, BJ, DB, DJH, RS, FG, and FR designed research studies. AA, JB, NFB, ADB, JA, BJ, AT, OW, JL, KE, CC, and EO conducted experiments and analyzed data. NI, NH, JC, and RS provided reagents. AA, FG, and FR wrote the manuscript. All authors contributed to editing the manuscript.

## Supplementary Material

Supplemental data

Supplemental data set 1

## Version 1 05/22/2023

Electronic publication
