# Supplementary material for: Hypothalamic and brainstem glucose-dependent insulinotropic polypeptide receptor neurons employ distinct mechanisms to affect feeding
Source: JCI Insight. 2023 May 22;8(10):e164921. doi: 10.1172/jci.insight.164921 (PMC10322681; doi:10.1172/jci.insight.164921)
Supplement: Supplemental data [file jciinsight-8-164921-s219.pdf]

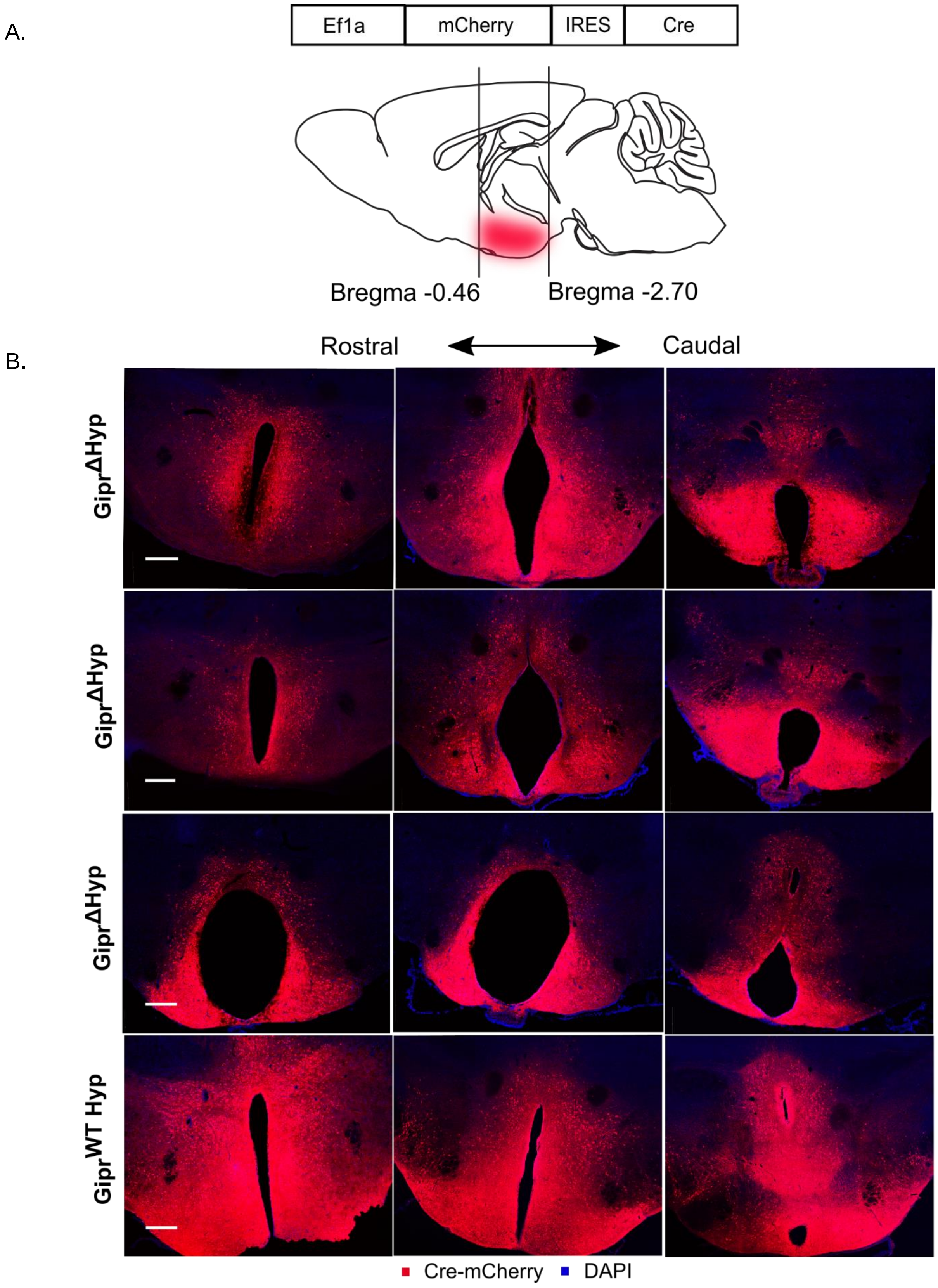

Supplementary Figure 1. Targeting confirmation for hypothalamic-specific knock down of *Gipr*. *Gipr*$^{fl/fl}$ or *Gipr*^{WT} controls received stereotaxic injection of AAV particles packaging a construct encoding Cre recombinase and an mCherry fluorescent label into the hypothalamus to produce *Gipr*$^{\Delta Hyp}$ and *Gipr*$^{WT\ Hyp}$ mice, respectively. *Gipr*$^{\Delta Hyp}$ and *Gipr*$^{WT\ Hyp}$ mice were placed on high fat diet until obese prior to treatment with vehicle, GLP-140 (30 nmol/kg, sc), or GLP-140 (30 nmol/kg, sc) + GIP-085 (300 nmol/kg sc) for 12 days. At the end of the study mice were perfuse-fixed, and their brains were isolated. Serial coronal brain sections were stained for mCherry to assess rostral-caudal viral spread (**A**), and to confirm hypothalamic targeting (**B**). Images are representative of staining from 6 *Gipr*$^{\Delta Hyp}$ mice and 3 *Gipr*$^{WT\ Hyp}$ mice. Scale bars are 300 µm.

A. *Gipr* $^{+/+}$ Cre $^{-/-}$

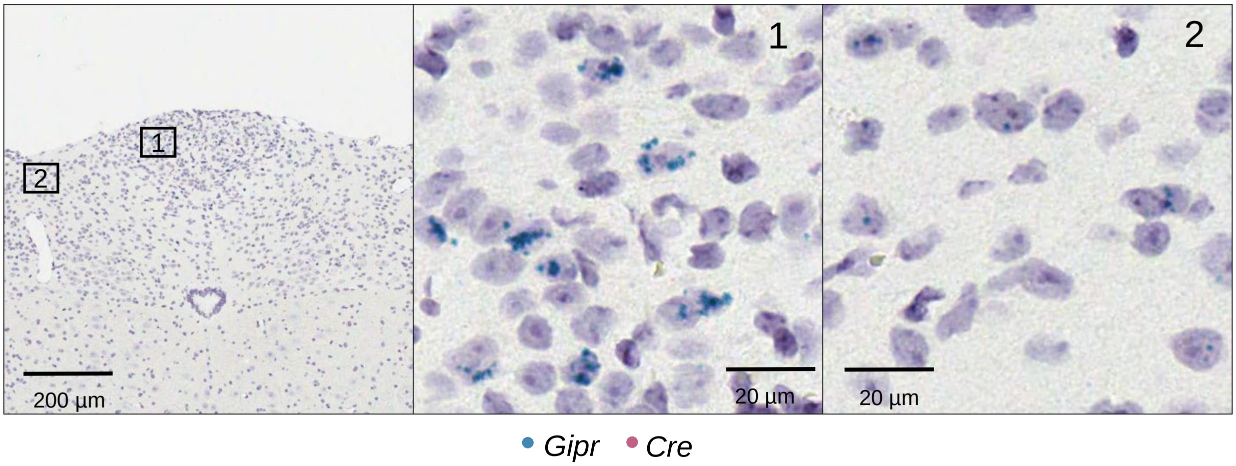

B. *Gipr* $^{+/-}$ Cre $^{+/-}$

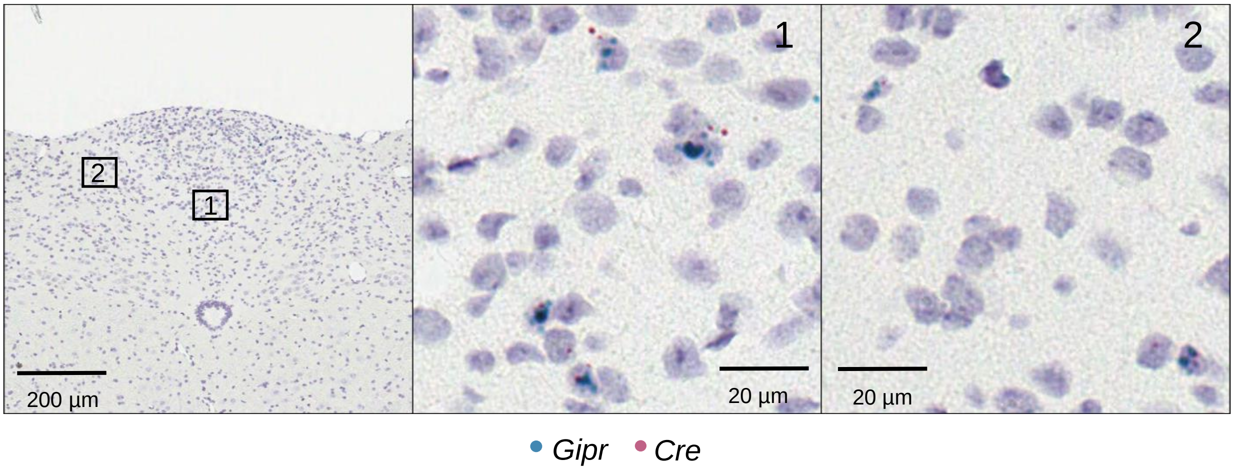

Supplementary Figure 2 *In situ* hybridization of *Gipr* and *iCre* in *Gipr*-Cre mice. Dual-label chromogenic *in situ* hybridization showing co-localisation of *Gipr* (cyan) with iCre (red) transcript in brain stem tissue isolated from *Gipr*$^{+/+}$ Cre$^{-/-}$ (**A**) or *Gipr*$^{+/-}$ Cre$^{+/-}$ (**B**) mice. Nuclei are counterstained with nissl (blue). Photomicrographs are representative from n = 3 mice per genotype.

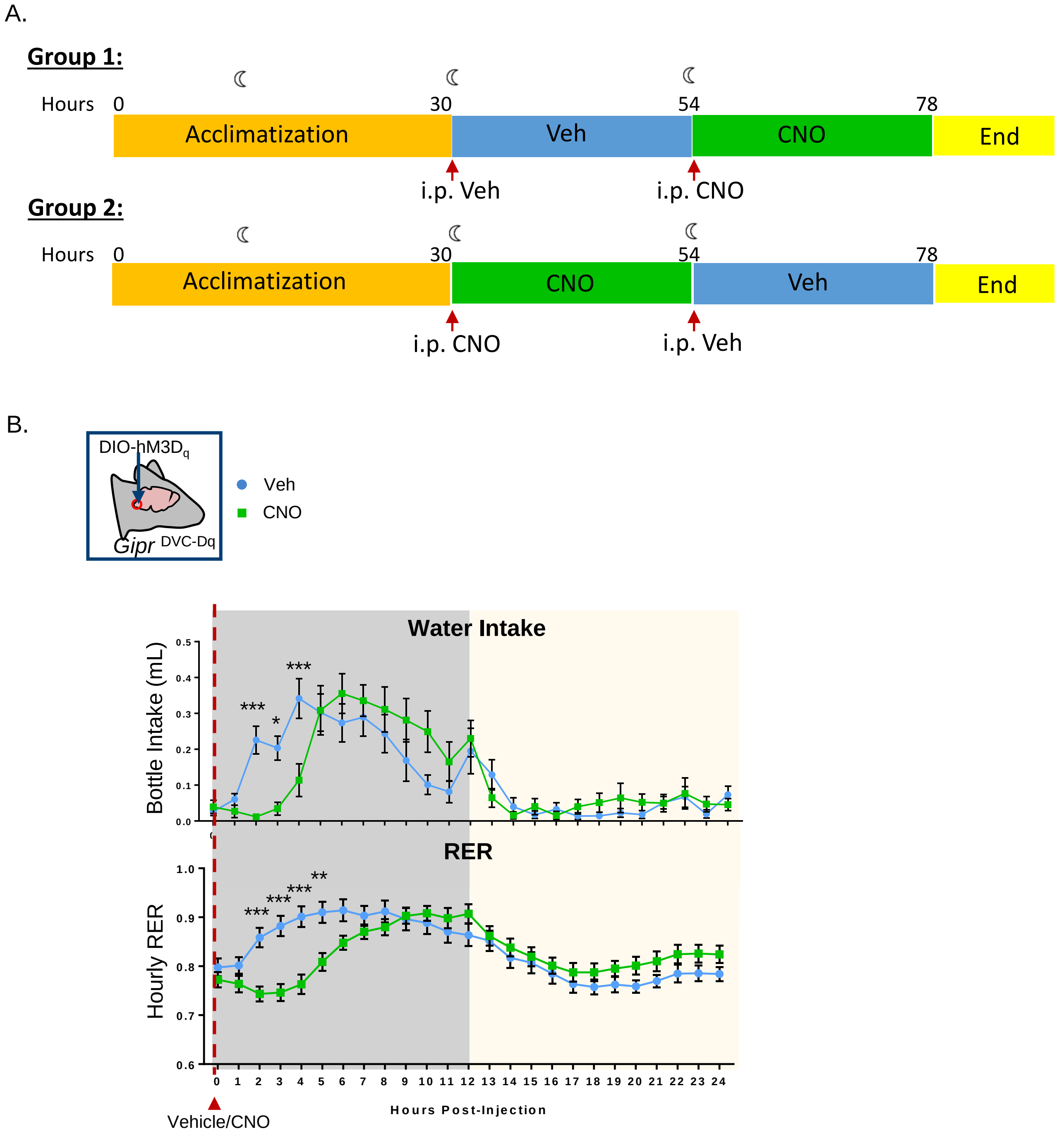

Supplementary Figure 3: A. Diagram outlining crossover study design. B. Acute activation of *Gipr* neurons in the DVC suppresses water intake and RER. *Gipr*-Cre mice were injected with AAV-*hSyn*-DIO-hM3D(Gq)-mCherry into the DVC to produce *Gipr*$^{DVC\text{-}Dq}$ mice. Mice were housed in indirect calorimetry cages equipped with continuous monitoring. CNO (1 mg/kg) or vehicle was injected IP at the onset of the dark phase. Mice were given standard chow and drinking water. Data are plotted as means +/- SEM. Statistical comparisons made using a repeated measures 2-way ANOVA with a Sidak's post-hoc test. * $p < 0.05$, ** $p < 0.01$, *** $p < 0.001$; n = 16.

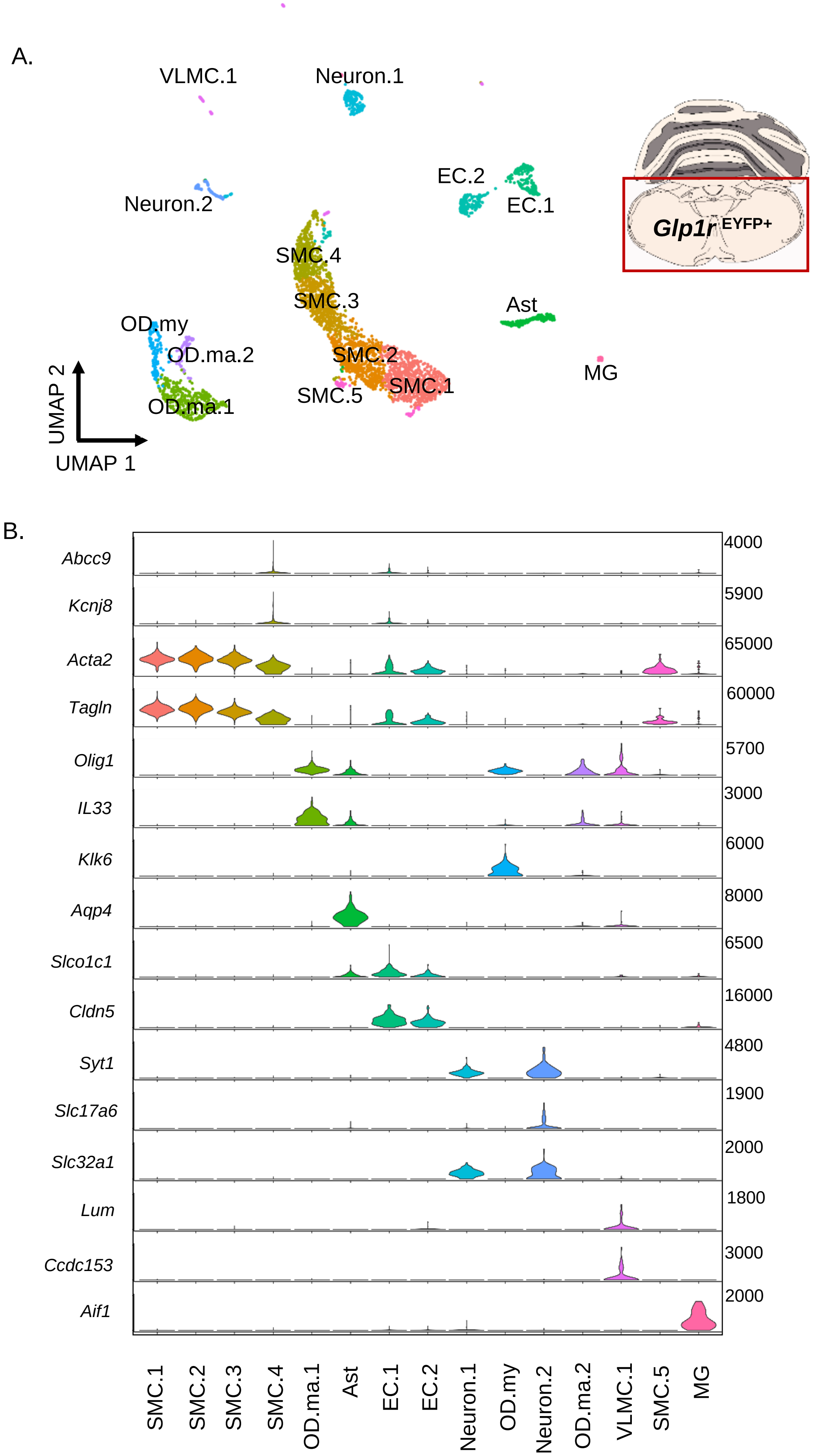

Supplementary Figure 4. Transcriptomic characterisation of *Glp1r* expressing cells in the hindbrain. *Glp1r* cells were isolated from single-cell digests of hindbrain sections from *Glp1r*^{EYFP} mice via FACS, and their transcriptomes were characterised via scRNAseq followed by clustering analysis. **A**. UMAP visualisation of *Glp1r*^{EYFP} cells. Cell types were assigned according to expression of marker genes (SMC.1, SMC.2, SMC.3, SMC.4, SMC.5 = smooth muscle cells; Ast = astrocytes; EC.1, EC.2 = endothelial cells; OD.ma.1, OD.ma.2 = mature ODs; OD.my = myelinating ODs; Neuron.1, Neuron.2 = neurons; VLMC.1 = vascular leptomenigeal cells; MG = microglia) (**B**).

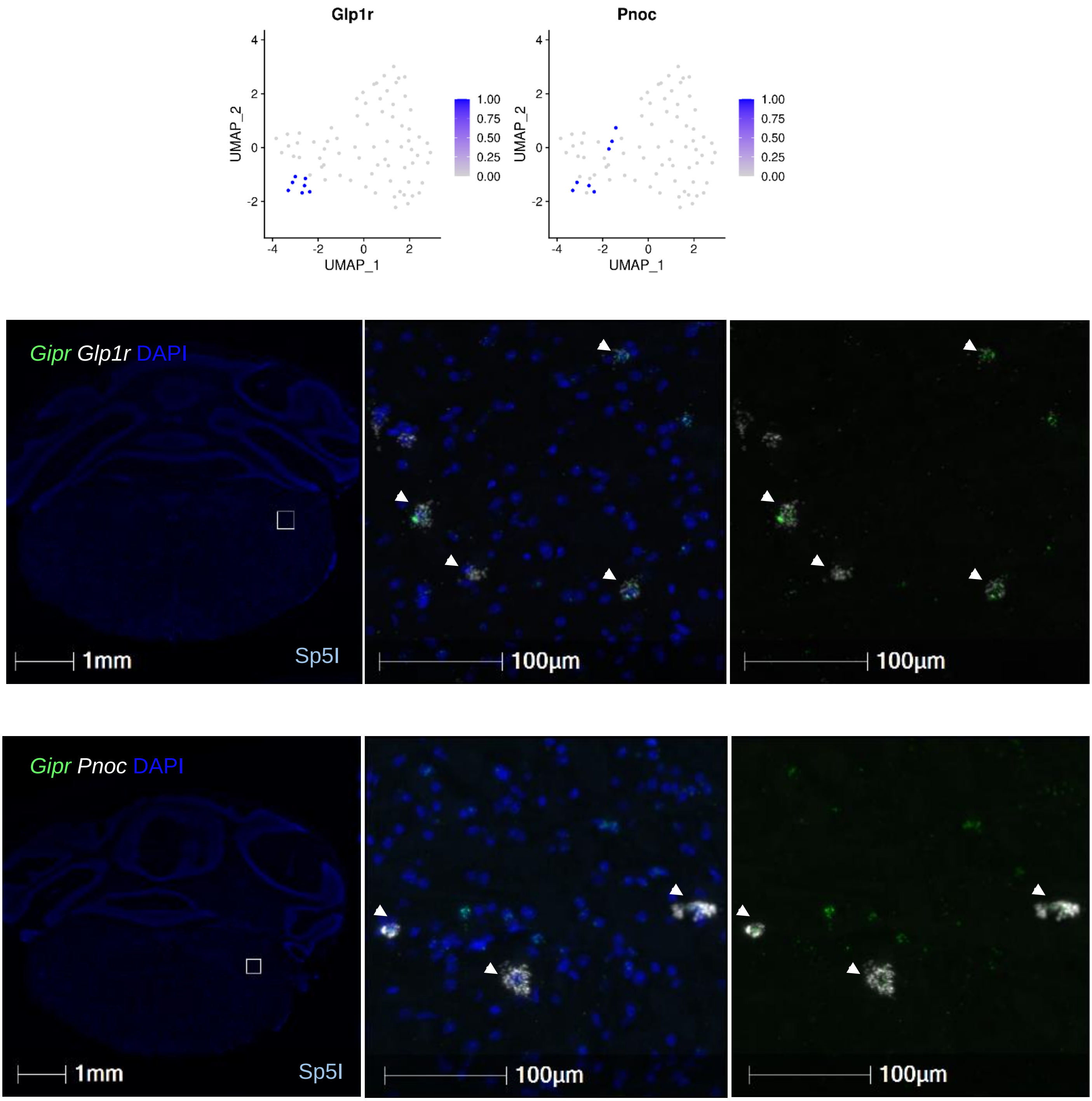

Supplementary Figure 5 *Gipr*/*Glp1r* co-expressing cells of the Sp5I. UMAP plots showing cells with raw UMI > 1 for *Glp1r* or *Pnoc* in scRNAseq data from *Gipr*^{EYFP} neurons. Dual-label fluorescent *in situ* hybridization showing co-localisation of *Gipr* with either *Glp1r*, or *Pnoc* transcript in the Sp5I of brain stem tissue from C57BL/6 mice. Nuclei are counterstained with DAPI (blue). Photomicrographs are representative of experiments conducted in tissue from n = 3 mice.

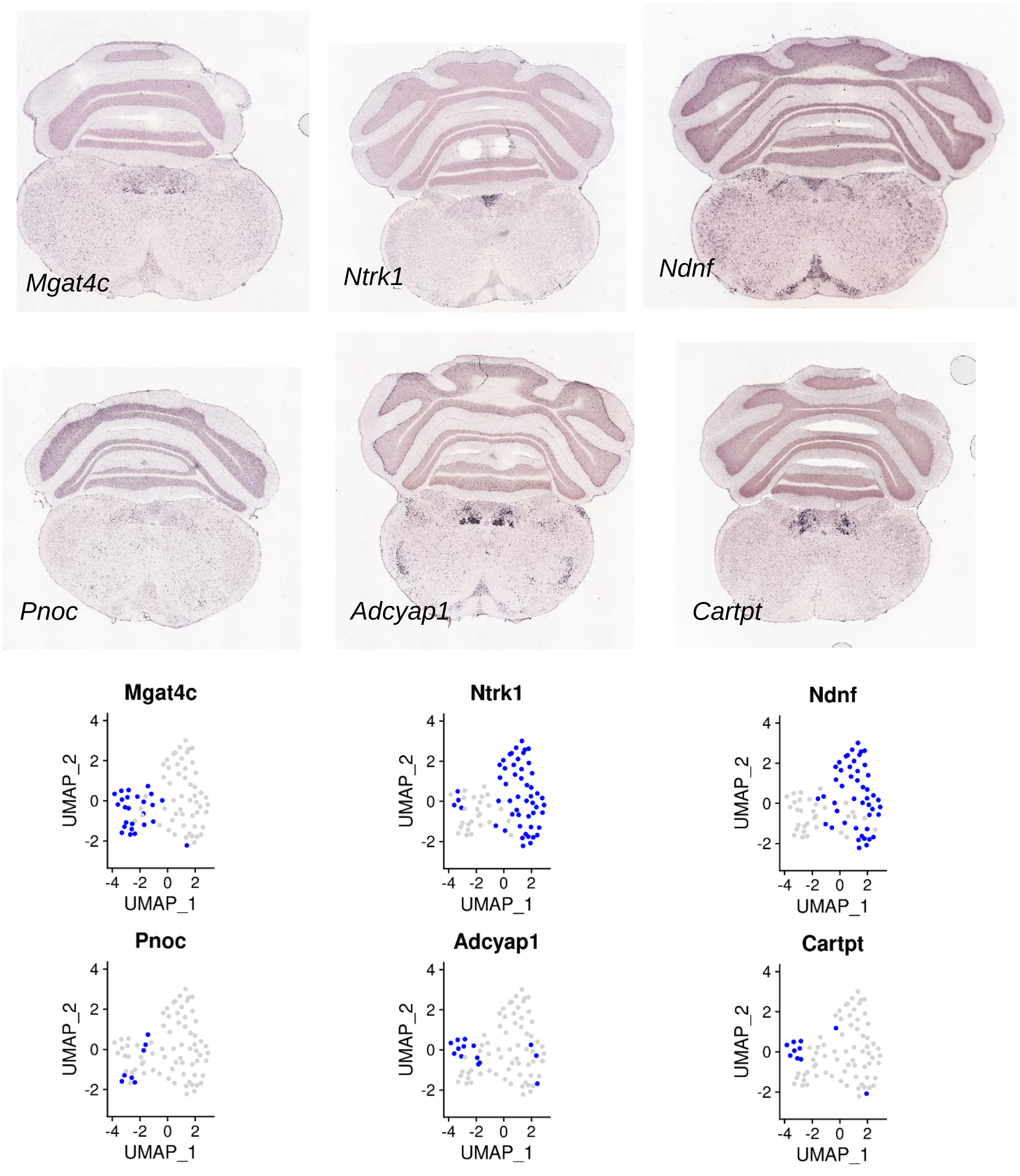

Supplementary Figure 6 Regional markers for hindbrain nuclei. Cluster-specific markers for *Gipr*EYFP neurons were identified using Wilcoxon rank-sum testing, compared to published brain region-specific transcriptional markers, and mapped to specific hindbrain nuclei using the Allan Brain Atlas. Feature plots show selected cluster marker expression on UMAP of *Gipr*EYFP neurons. Representative ISH images are from the Allen Brain Atlas.

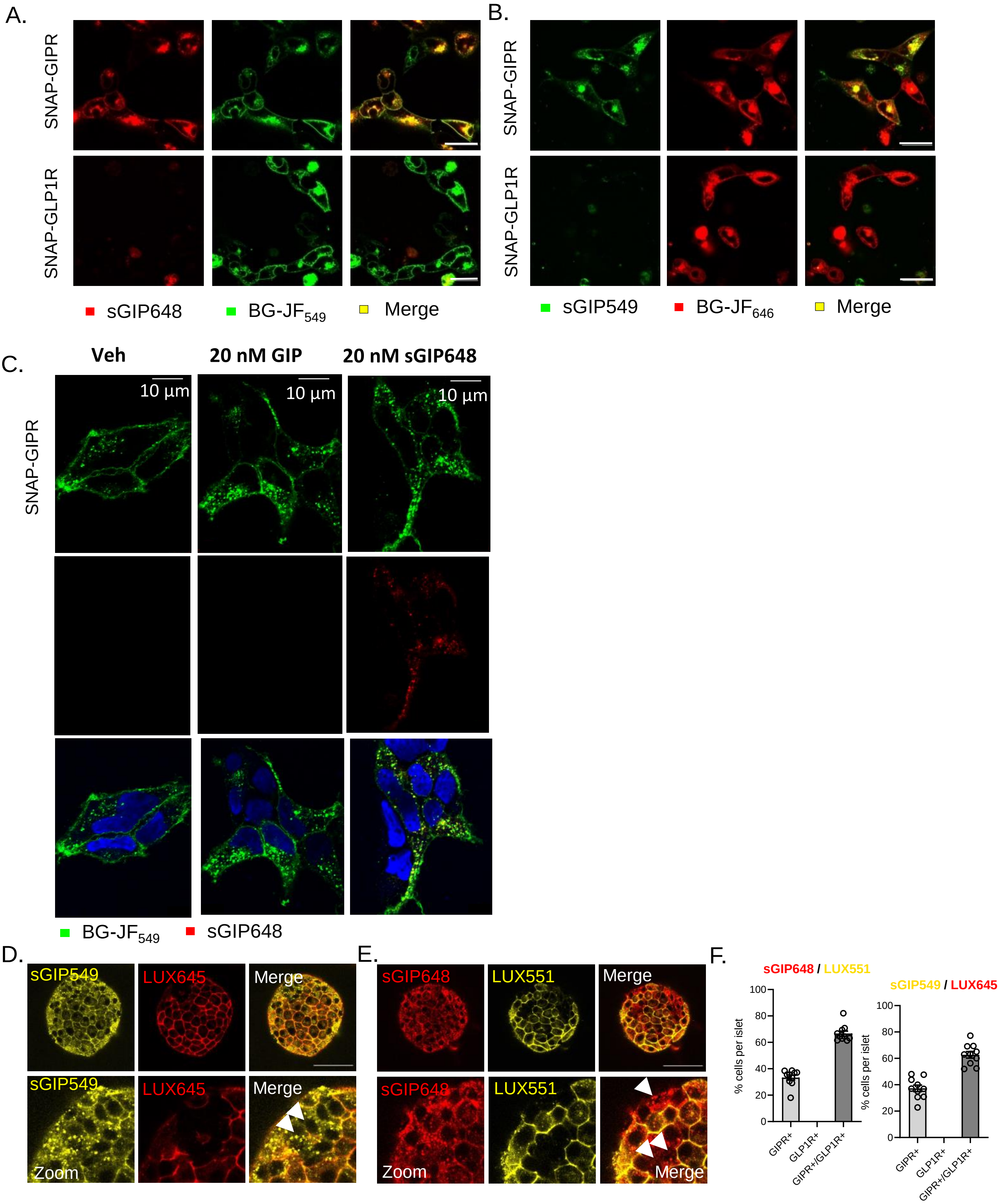

Supplementary Figure 7. **A.** sGIP648 labels SNAP-GIPR-AD293, but not SNAP-GLP1R-AD293 cells, orthogonally labelled with the cell-permeable SNAP label BG-JF$_{549}$. **B.** As for A, but using sGIP549 and BG-JF$_{646}$. A-B Images are representative of n = 3 experiments. Scale bar = 18 µm **C.** SNAP-GIPR internalisation after 60 min treatment with 20 nM GIP(1-42), sGIP648 or vehicle in T-REx-SNAP-GIPR cells. Images are representative of n = 2 experiments. **D-F.** sGIP549 (D) and sGIP648 (E) label GIPR$^+$ cells (presumed alpha cells, which are more abundant at the islet surface) and GIPR$^+$ GLP1R$^+$ cells (presumed to be beta cells, since alpha cells do not express *Glp1r*). As expected, all GLP1R$^+$ cells are GIPR$^+$. n= 10 islets for each sGIP549/LUX645, sGIP648/LUX551 co-labelling quantification(F). Arrows show cells that express only GIPR. Scale bar = 53 µm. Bar graphs shown mean ± SEM.

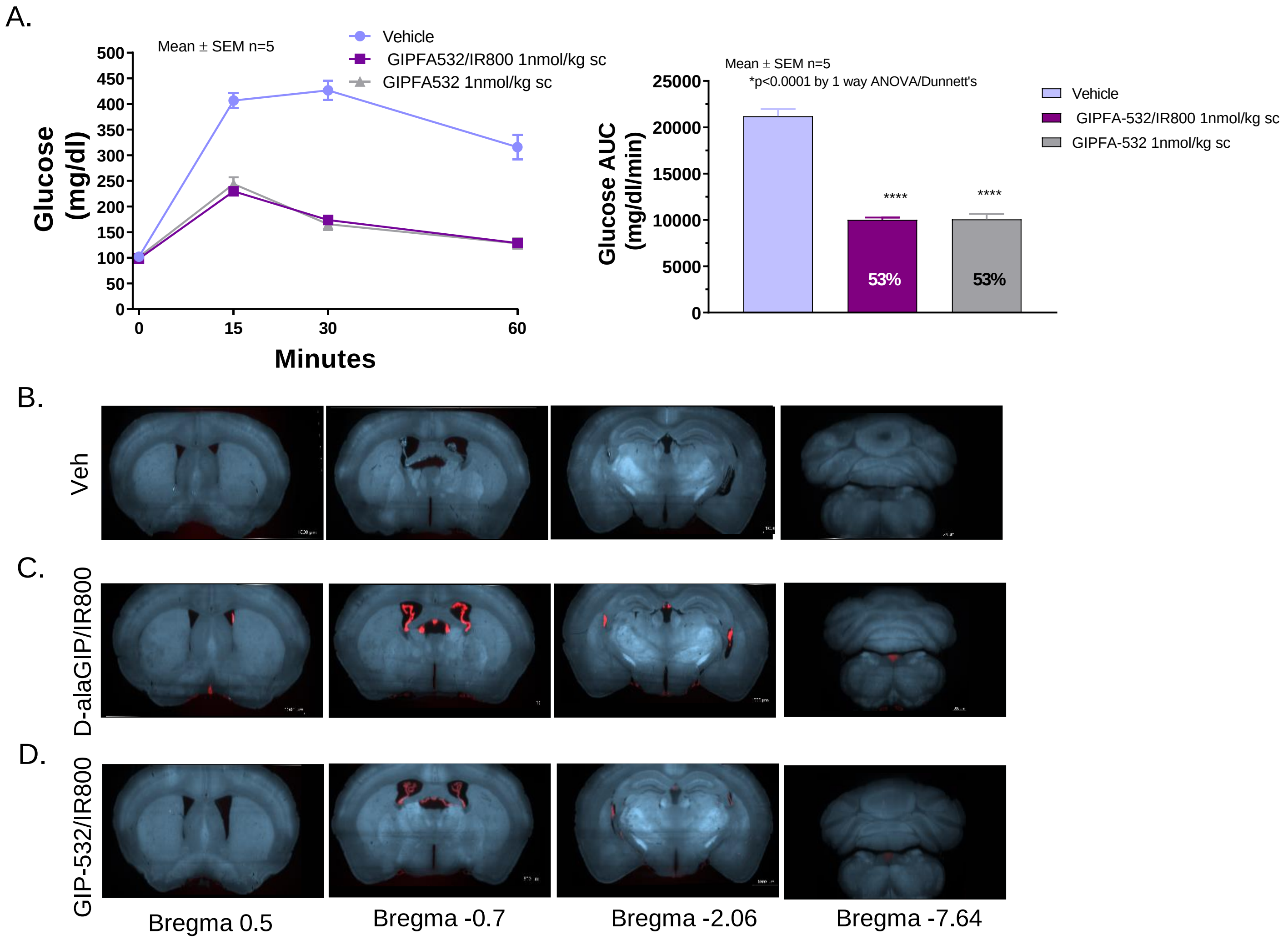

Supplementary Figure 8. GIP-532/IR800 enhances glucose tolerance *in vivo* and localises to circumventricular organs. **A**. C57BL/6 mice were treated with vehicle, GIP-532 (1 nmol/kg, sc), or GIP-=532/IR800 (1nmol/kg sc) 30 min prior to an intraperitoneal glucose tolerance test. Data are presented as mean +/- SEM. *** p < 0.001 compared with vehicle. Statistical analysis performed using a one-way ANOVA, followed by Dunnett's multiple comparisons test. n = 5 mice for each drug treatment. B. C57BL/6 mice were treated with vehicle (**B**), D-alaGIP/IR800 (100 nmol/Kg) (**C**) or GIP-532/IR800 (100 nmol/Kg sc) (**D**) 2 hr prior to tissue harvest. Brains were cleared via iDISCO protocols. Labelled peptide distribution was revealed using light sheet imaging. Photomicrographs are representative of imaging data from n = 4 mice per treatment.

Supplementary Methods and Materials

Animals

All mice were group-housed and maintained under SPF health/immune status in individually ventilated cages with standard bedding and enrichment unless otherwise stated. Mice were housed in a temperature (20-22 ºC) and humidity-controlled room on a 12h/12h light/dark cycle with *ad libitum* access to water and standard laboratory chow diet, unless otherwise stated.

Gipr-Cre (1) or *Glp1r*-Cre (2) mice were crossed with a *ROSA26*-EYFP or a *ROSA26*-GCaMP3 reporter strain (3) to enable fluorescent detection of cells expressing *Gipr* or *Glp1r*. In studies using global *Gipr*$^{-/-}$ mice, *Gipr*-Cre mice were bred to homozygosity for Cre as previously described (1). Otherwise all *Gipr*-Cre animals used in circuit tracing and *in vivo* phenotyping studies were heterozygous for both *Gipr* and Cre. *Gipr*$^{fl/fl}$ mice used in central studies were a kind gift from N. Inagaki (Kyoto University, Kyoto, Japan) (4). *Sst*-Cre (5) and *Gipr*$^{fl/fl}$ strains were crossed to produce *Sst*-Cre^{+}/*Gipr*$^{fl+}$ mice (knock out of *Gipr* in *Sst*-expressing cells) with *Sst*-Cre^{-}/*Gipr*$^{fl-}$ littermate controls. To specifically knock out *Gipr* in the hypothalamus, *Gipr*$^{fl/fl}$ mice were stereotaxically injected with AAV viral vectors encoding Cre recombinase, producing *Gipr*$^{Hyp\ KO}$ or *Gipr*$^{Hyp\ WT}$ controls. *Mip-Cre* and *Gipr*$^{fl/fl}$ strains (6) were bred to produce *Mip-Cre*$^{+/-}$*;Gipr*$^{fl/fl}$ (*Gipr*$^{-/-\beta cell}$, beta cell Gipr KO) and *Mip-Cre*$^{+/-}$*;Gipr*$^{+/+}$ (*Mip*-Cre, littermate control) mice. Cre lines and reporter strains were on a mixed C57B6J/N genetic background.

Long-acting GIPR and GLP-1R agonist peptide synthesis

Long-acting agonists for GIPR (GIP-085) and GLP-1R (GLP-140) were synthesized at Eli Lilly and Co (Indianapolis, Indiana) as previously described (7, 8).

Fluorescent peptidic agonist synthesis

Stabilized GIP (sGIP) peptides were synthesized on solid phase support before global deprotection, purification by reverse-phase HPLC and HRMS characterization. sGIP was conjugated to a red (sGIP549) and a far-red (sGIP648) fluorophore using “click chemistry” and, after HPLC purification, characterized by LCMS and HRMS. Amino acid sequence and molecular structures are submitted for patent filing and will be disclosed in a future publication.

Labelled short-acting GIPRAs peptides (GIP-532/IR800 and d-AlaGIP/IR800) were synthesized at Eli Lilly and Company (Indianapolis, Indiana).

AD293 culture and labelling

AD293 cells were cultured in growth medium (DMEM D6546 supplemented with 10% FBS, 1% penicillin/streptomycin, 2 mM L-glutamine) at 37 °C, 5% CO_2. Cells were seeded into 96-well glass-bottom plates (E0030741013, Eppendorf) previously treated with 0.01% Poly-L-lysine solution (Merck). The next day at confluency 80-90%, cells were transfected using 0.3 µL of Lipofectamine 2000 (Invitrogen) and 50 ng of plasmids SNAP-GLP1R (Cisbio) or SNAP-GIPR in OptiMEM per well. Cells were labelled the next day in growth media supplemented with 100 nM sGIP549 and 100 nM of the antagonist GLP1R probe LUXendin645 (LUX645) or 100 nM sGIP648 and Luxendin551 (LUX551) for 60 min at 37 °C, 5% CO_2. After one wash, cells were imaged in growth media.

T-REx-GIPR cell culture and labelling

A custom GIPR expression plasmid pcDNA5-SNAP$_f$-GIPR-SmBiT was generated by Genewiz (Leipzig, Germany) to the following design: codon-optimised wild type human GIPR was tagged at the N-terminus with a fast-labelling SNAP$_f$ tag and upstream signal peptide based on that of the 5-HT$_{3A}$ receptor (MDSYLLMWGLLTFIMVPGCQA), plus C-terminal SmBiT tag, and inserted into the pcDNA5/FRT/TO expression vector. T-REx-SNAP-GIPR cells were generated from Flp-In™ T-REx™ 293 cells (Thermo Fisher) by co-transfection with pOG44 (Thermo Fisher) and pcDNA5-SNAP$_f$-GIPR-SmBiT in a 9:1 ratio, followed by selection with 100 µg/mL hygromycin. The resulting cell line was maintained in DMEM supplemented

with 10% FBS and 1% penicillin/streptomycin. To observe GIPR internalisation, cells were seeded on poly-D-lysine-coated coverslips in complete medium ± 0.1 µg/mL tetracycline for 24 hours to induce SNAP-GIPR expression. SNAP-GIPR labelling was performed with 0.5 µM SBG-JF_{549} for 10 min before treatment with 20 nM GIP(1-42) or sGIP648 for 60 min in complete medium before PFA fixation. Images were taken with a Zeiss LSM780 laser scanning confocal microscope with a 63x/1.4 NA oil immersion objective using the same laser intensities for the different conditions.

cAMP assay

T-REx-SNAP-GIPR cells, pre-treated with 0.1 µg/mL tetracycline for 24 hours to induce SNAP-GIPR expression, were stimulated with GIP(1-42), sGIP549 or sGIP648 for 30 min at 37°C before lysis. cAMP was assayed by HTRF (Cisbio cAMP Dynamic 2).

Islet isolation, culture and labelling

Tissue from 8-12 week old *Mip*-Cre$^{+/-}$;*Gipr*$^{fl/fl}$ (*Gipr*$^{-/-\beta cell}$), *Gipr*$^{fl/fl}$, *Gipr*-Cre *ROSA26*-GCaMP3 (*Gipr*^{GCaMP3}) or C57BL6 mice was used. Mice were euthanized by rising CO_2 and cervical dislocation, before bile duct injection and inflation of the pancreas through the pancreatic duct with either collagenase V (0.7 mg/ml) in Hanks' balanced salt solution or 1 mg/ml SERVA NB8 collagenase (Amsbio, Catalog # 17456.01) in RPMI. The pancreas was then excised and digested for 10-12 min at 37 °C before either hand-picking or gradient separation of islets using Histopaque. Islets recovered overnight prior to all experiments. Islets were maintained at 37°C and 5% CO_2 in RPMI medium containing 10% FCS, U/mL penicillin, and 100 µg/mL streptomycin.

For fluorescent peptide labelling, islets were incubated for 60 min at 37 °C with either 100 nM sGIP549 + 100 nM LUX645 or 100 nM sGIP648 + 100 nM LUX551, followed by three washes. Imaging was performed using Zeiss LSM780/LSM880 meta-confocal microscopes equipped with sensitive GaAsP spectral detectors and a 40x/1.2 W Korr FCS M27 objective. Excitation (ex) and emission (em) wavelengths were as follows: sGIP549 and LUXendin551,

ex λ = 568 and em λ = 569-623; sGIP648 and LUXendin645, ex λ = 633 and em λ = 641-694. Linear adjustments to brightness and contrast were applied to representative images, with intensity values maintained between samples to allow cross-comparison.

Brain labelling

Mice were injected intravenously with vehicle or sGIP648 (100 pmol/g), or subcutaneously with vehicle, GIP-532/IR800 (100 nmol/Kg), or D-alaGIP/IR800 (100 nmol/Kg). Mice were terminally anaesthetised and transcardially perfused with 0.1M PBS followed by 4% PFA. Brains harvested from sGIP648-treated animals were serially sectioned at 30 µm and mounted on slides before imaging. Images were taken with a Leica TCS SP8 laser scanning confocal microscope with a 40x/1.4 NA oil immersion objective using the same laser intensities for the different conditions.

For brains harvested from GIP-532/IR800- or D-AlaGIP/IR800-treated animals, male 7-8-week-old lean C57BL/6JRj mice, fed regular chow diet (Altromin 1324), were randomized by body weight into 4 groups (n=6/group) for acute subcutaneous compound dosing with either vehicle, 100 nmol/kg dAla2GIP, 30 nmol/kg GIP-532 or 100 nmol/kg GIP-532. Mice were terminated 2 h following treatment. In brief, mice were terminally anaesthetised and transcardially perfused with heparinized (15,000 IU/l) phosphate buffered saline (PBS) for 2 minutes at a rate of 20-25 ml/min, followed perfusion for 5 minutes with 10% neutral buffered formalin (NBF, CellPath Ref. 1000.5000) at the same perfusion rate. Brains, iWAT and BAT were carefully dissected, and immersion fixed in NBF overnight at room temperature. The samples were then washed 3×30 minutes in PBS with shaking.

Samples were processed in batch to minimize experimental variability. To achieve good tissue morphology, the samples were dehydrated twice with a rehydration step in-between. First, the samples were washed 3×30 minutes in PBS with shaking and dehydrated in methanol/H_2O series: 20%, 40%, 60%, 80% and 100% for 1 h each at room temperature, followed by incubation in 100% methanol overnight. Next day, the samples were washed in

80%, 60%, 40% and 20% methanol in 2% Triton X-100 in PBS for 1 h each at room temperature. Samples were further washed for 1 h in 2% Triton X-100 in PBS and overnight in 2% Triton X-100 in PBS. Samples were dehydrated again in methanol/H_2O series: 20%, 40%, 60%, 80% and 100% for 1 h each at room temperature, followed by overnight incubation in 100% methanol. Next day, they were incubated for 3 h in 66% DCM (Dichloromethane)/33% methanol at room temperature and in 100% DCM twice for 15 min (with shaking). The samples were finally transferred to dibenzyl ether (DBE) and stored in closed glass vials in dark.

Brains were imaged in DBE using Lavision light-sheet ultramicroscope II (Miltenyi Biotec GmbH, Bergisch Gladbach, Germany) with Zyla 4.2PCL10 sCMOS camera (Andor Technology, Belfast, UK), SuperK EXTREME supercontinuum white-light laser EXR-15 (NKT Photonics, Birkerød, Denmark) and MV PLAPO 2XC (Olympus, Tokyo, Japan) objective. Brains were mounted with ventral side up to a silicone-casted sample holder with neutral silicone gel. ImSpector microscope controller software (v7) was used (Miltenyi Biotec GmbH, Bergisch Gladbach, Germany). Images were acquired at 0.63× zoom (1.2× total magnification) with 553 ms exposure time in a z-stack at 10 μm intervals. Horizontal focusing was captured in 9 planes with blending mode set to the centre of the image to merge raw images. Autofluorescence images were captured at 560±20 nm (excitation) and 628±20 nm (emission) wavelength (80% laser power in ImSpector software, 100% NKT laser). Compound fluorescence was imaged at 785±12 nm excitation wavelength and 845±27 nm emission wavelength (100% laser power in software, 100% NKT laser). Adipose samples were imaged similarly to brains, except that single-sided illumination at 3.2× zoom (6.4× total magnification) was used and images were acquired at 3 μm intervals, using horizontal focus blending over 7 planes.

Image analysis was carried out in Python 3.7. Fluorescence signal from individual 3D imaged brains were transferred to average mouse brain atlas template (9), enabling to quantify accumulated fluorescence in individual brain regions. Group average fluorescence intensities in brain regions were compared to background signal in the Vehicle group. 3D images and 2D

views were collected from adipose tissue samples. Bitplane Imaris software 2 (Oxford instruments, Abington, UK) was used for 3D reconstructions and movies.

Immunohistochemistry

For immunohistochemistry performed in brain slices, animals were terminally anaesthetised with sodium pentobarbital (200 mg/kg) and transcardially perfused with 0.1 M PBS followed by 4% PFA. Brains were extracted and post-fixed in 4% PFA for 24 h at 4 °C then transferred to 30% sucrose solution at 4 °C for 48 h. Brains were sectioned coronally at 30 μm using a freezing microtome and stored in cryoprotectant medium. For immunofluorescent staining, slices were washed in PBS, prior to blocking for 1 h in 5% normal serum then incubation with primary antisera in blocking solution overnight at 4 °C (GFP Abcam 13970, 1:1000; DsRed Takara Bio 632496, 1:1000; c-Fos Synaptic Systems 226 003, 1:1000). Slices were washed in and incubated with the appropriate secondary antisera (Alexa Fluor® 488 or 555; Invitrogen A-11039, A-11008, A-27039) diluted 1:500 for 2 h at room temperature. Following washing, mounted sections were coverslipped on superfrost slides using Vectashield (Vector Laboratories). Slides were imaged using a Zeiss Axio Scan.Z1 slide scanner with a 20x air objective (Zeiss) or Leica TCS SP8 laser scanning confocal microscope with a 40x/1.4 NA oil immersion objective using the same laser intensities for the different conditions. Images were analysed in Halo (Indica Labs), Zen (Zeiss) and ImageJ image analysis software.

RNAscope *in situ* hybridization in brain slices

RNAscope in situ hybridization studies were conducted using RNAscope technology (Advanced Cell Diagnostics, Newark CA).

Fluorescent detection:

C57BL6J mice were terminally anesthetized either by overdose of isofluorane or terminal i.p. injection of sodium pentobarbital (200 mg/kg) and whole brains were collected, snap frozen on crushed dry ice. Each brain is divided into forebrain and hindbrain by a coronal

cut at the level of the pons and mounted on the pre-cooled cryostat holder with Tissue-Tek O.C.T. compound (Sakura Finetek). Series of 12 µm thick coronal sections covering the AP and NTS are cut on a cryostat and collected on microscope slides. 2 sections from AP/NTS in 3 mice were collected for each hybridization experiment. Slides are stored at –80°C until further use. Sections were hybrized with RNAscope multiplex probes as indicated (mouse: *Gipr* 319121, *Glp1r* 418851, *Syt1* 491831, *Pnoc* 437881, *Slc17a6* 319171, *Cartpt* 432001, *Th* 317621, *Penk* 318761, *Npy2r* 315951, *Oxtr* 402651) according to the manufacturer's instructions. Sections were counterstained with DAPI to mark nuclei, cover slipped, and imaged using an Olympus VS120 Fluorescent Scanner. Quantification of mRNA co-localisation was evaluated manually using HALO image analysis software (Indica Labs). Tissue sectioning, RNAscope hybridization, and slide scanning were performed at Gubra (Horsholm, Denmark).

Chromogenic detection:

Brain tissue was collected from *Gipr*$^{+/+}$ *Cre*$^{-/-}$ and *Gipr*$^{+/-}$ *Cre*$^{+/-}$ mice (*n* =3 for each genotype). Animals were terminally anaesthetized with pentobarbital and transcardially perfused with 0.1M PBS followed by 10% formalin. Brains were extracted and post-fixed for 24 h before paraffin embedding. Series of 5 µm thick coronal sections covering the AP and NTS are cut on a cryostat and collected on microscope slides. One section from the AP/NTS region from each of three mice for each genotype was collected and onto microscope slides. Sections were hybridized with RNAscope multiplex probes (mouse: *Gipr* 319128, *iCre* 423328) according to the manufacturer's directions. Slides were imaged using a Leica Aperio ImageScope slide scanner with a 20x objective.

RNAscope In Situ Hybridization Nodose Ganglia

Nodose ganglia were collected from four C57BL/6 mice (2M/2F, 12-20wks) for RNAscope analysis. Animals were anaesthetized with pentobarbital (200 mg/kg) and transcardially perfused with heparinized PBS followed by 4% PFA in PBS. Nodose ganglia

were extracted and post-fixed in 4% PFA for 2 hours before being transferred to 30% sucrose for 24 hours at 4°C. Coronal sections were cut at 10µm on a Leica CM3050s cryostat and collected on Superfrost Plus slides for storage at -80°C until required.

Simultaneous detection of *Gipr*, *Glp1r* and *Oxtr* was performed on fixed, frozen nodose ganglia sections using the RNAscope Multiplex Fluorescent Kit V2 (Advanced Cell Diagnostics). RNAscope in situ hybridization was performed as per the manufacturer's instructions, with a modification of the pretreatment procedure (Protease III incubation conducted for 30 min at 40°C) providing optimal signal detection of the target mRNAs. Probes for *Gipr* (#319121), *Glp1r* (#418851-C2) and *Oxtr* (#412171-C3) and positive (Ubc) and negative (DapB) controls were hybridized and labelled with the following fluorophores: TSA Vivid 520 (C2 and controls), TSA Vivid 570 (C3 and controls) and TSA Vivid 650 (C1 and controls). This was followed by additional processing for immunofluorescent labelling of the neuronal marker HuC/D. Slides were incubated overnight with anti-HuC/D mouse primary antibody (A21271, Thermo Fisher Scientific; 1:100 in PB containing 1% normal goat serum and 0.1% Triton X-100) followed by 2 h in goat anti-mouse Alexa Fluor 405 secondary antibody (A48255, Thermo Fisher Scientific; 1:400 in PB containing 1% normal goat serum and 0.1% Triton X-100). Slides were then cover slipped using Vectashield HardSet mounting medium (Vector labs).

Slides were imaged on an EVOS M7000 multichannel epifluorescence imaging system (Invitrogen). Multichannel z-stack images were captured at 40x. Image acquisition parameters were set using the positive and negative control probes for each fluorophore as reference. Images were tiled and used to generate multichannel maximum intensity projection z-stacks using EVOS software. Images were processed for publication using Fiji image analysis software.

Quantitative PCR

Quantitative RT-PCR was performed on whole nodose ganglia (NG). Tissue was isolated from 12-18 week old female C57BL6 mice. 5-6 mice were pooled for each NG sample.

NG were isolated into RLT lysis buffer (QIAGEN) before dounce homogenisation. Total RNA was extracted using RNeasy Plus Micro kit (QIAGEN) according to the manufacturer's protocol. DNAse1 treatment was performed using gDNA spin columns (QIAGEN). RNA was reverse transcribed using the SuperScript IV Reverse Transcriptase (Thermo Fisher Scientific).

qPCR was performed with a QuantStudio 5 Real-Time PCR system (Applied Biosystems). The PCR reaction mix consisted of first-strand cDNA template, TaqMan™ gene expression primer/probe mix (Thermo Fisher Scientific; *Gipr* mm01316344_m1, *Glp1r* mm00445292_m1, *Actb* mm02619580_g1), and PCR master mix (Thermo Fisher Scientific). Expression of the selected targets was compared to that of *Actb* measured on the same sample in parallel on the same plate, giving a CT difference (ΔCT) for *Actb* minus the test gene. Data were converted to relative expression levels (2ΔCT) for presentation in the figures.

Flow Cytometry

Single cell suspensions were prepared from coronal sections of hindbrain tissue pooled from four to six *Gipr*^{EYFP} or *Glp1r*^{EYFP} male mice that were four to six weeks old as described previously (1). Tissue from the brain stem located between -6.00 to -8.12 mm relative to Bregma was dissected into Hibernate-A medium without calcium (BrainBits). The tissue was digested with 20 U/ml Papain (Worthington) for 30 min at 37°C, followed by trituration in Hibernate-A medium (Thermo Fisher Scientific) containing 0.005% (w/v) DNase 1 (Worthington). The cell suspension was filtered through a 40 µm cell strainer into a fresh tube.

Fluorescence-activated cell sorting was performed using a BD Influx Cell Sorter (BD Biosciences, Franklin Lakes, NJ, USA) to isolate *Gipr*$^{EYFP+}$ and *Glp1r*$^{EYFP+}$ cells prepared from *Gipr*^{EYFP} or *Glp1r*^{EYFP} mice, respectively. Cells were gated according to cell size (FSC), cell

granularity (SSC), FSC pulse-width for singlets, fluorescence at 488 nm/532 nm for EYFP and 647/670 nm for nuclear stain with DraQ5 (Biostatus).

Single Cell RNA Sequencing

Single cell cDNA libraries from purified *Gipr*$^{EYFP+}$ or *Glp1r*$^{EYFP+}$ cells were generated using the 10× Genomics Chromium Instrument and single-cell 3' Reagent kit (V3; 10X Genomics). Pooled libraries were sequenced on an Illumina NovaSeq 6000 instrument (28-bp first read, 91-bp second read). Library preparation and sequencing was performed by the Genomics Core, Cancer Research UK Cambridge Institute.

Sequencing reads were aligned to an amended annotation of the mouse genome (GRCm39). The 3' untranslated regions (UTRs) of *Glp1r*, *Ghsr*, *Prokr2* and *Prlhr* were compared against PolyA_DB v3 (Wang et al., 2018), and extended where necessary to include the furthermost 3' UTRs in keeping with previous studies (10-12). Downstream analyses on the unfiltered count matrices were performed using the Seurat v4 R package (13). Cells were filtered from the analysis if they contained fewer than 1000 RNA counts and fewer than 200 unique genes. There were a total of 5521 cells in the filtered *Gipr*$^{EYFP+}$ dataset, and 3835 cells in the filtered *Glp1r*$^{EYFP+}$ dataset.

Dimensionality reduction was performed on scaled, counts per million (CPM)-normalised data. Uniform manifold approximation and projection (UMAP) were generated using the top 11-15 principle components from principle component analysis (PCA). Clustering was performed using the Louvain algorithm. Gene markers for each cluster were determined using Wilcoxon's rank-sum test and were cross referenced against other bulk and single cell RNA sequencing databases RNAseq and in situ hybridisation (ISH) databases to assign cell type identities for each cluster (14-22).

For further analysis of neuronal clusters, neurons were selected based on raw UMI counts using the logic expression *Syt1* > 1 | *Snap25* > 1. Contaminating non-neuronal cells were filtered using *Mog* < 20, *Olig1*< 10, *Abcc9* < 12, and *Aqp4* < 2 for *Gipr*^{EYFP} neurons, with

additional filters for *Acta2* < 6 and *Tagln* < 5 for *Glp1r*^{EYFP} neurons. Filtered neurons were re-analysed for dimensionality reduction as described above.

To compare *Gipr*^{EYFP} and *Glp1r*^{EYFP} neurons, each respective dataset was normalised using the SCTransform function in Seurat prior to integration. Datasets were integrated using canonical correlation analysis (CCA), approximate nearest neighbours (ANN), and n = 2,000 integration features. Where differential expression analysis was performed a Wilcoxon's rank-sum test was implemented by Seurat's FindMarkers function.

Glucose Tolerance Tests

Lean 10 week-old male C57BL/6 mice were fasted overnight. Mice were dosed subcutaneously with GIP-532/IR800 (1 nmol/Kg), or vehicle 30 min prior to an intraperitoneal bolus of glucose (2 g/kg). Blood glucose concentrations were measured at 0, 15, 30, 60 and 120 min post glucose dose using glucometers. Data were used to generate area under the curve (AUC) calculations from the glucose concentrations measured between 0-120 min.

Viral Injections

Viral injections were performed in male and female *Gipr*-Cre or *Gipr*$^{fl/fl}$ mice aged between 8 and 16 weeks.

For viral injections into the hypothalamus and PBN, surgical procedures were performed under isofluorane anesthesia. Mice were stereotactically implanted with bilateral steel guide cannulae (Plastics One) positioned 1 mm above the target region. Stainless steel injectors (33 gauge, Plastics One) extending 1 mm from the tip of the guide were used for injections, delivering AAV at 75 nl/min. All coordinates relative to Bregma: MBH 500 nl A/P: −1.1 mm, D/V: −4.9 mm, M/L: +/- 0.4 mm bilateral, PVH 150 nl A/P: −0.9 mm, D/V: −3.7 mm, M/L: +0.2 mm unilateral, PBN 150 nl A/P: −5.0 mm, D/V: −2.5 mm, M/L: +1.35 mm unilateral. Animals received oral carprofen analgesia on the day of surgery and 3 days post-operatively. Mice were allowed a 2 week recovery period prior to commencing studies.

For viral injections into the DVC, mice were anaesthetized with ketamine (100 mg/kg)/ xylazine (10 mg/kg) and given carprofen analgesia (5mg/kg, sc). Mice were placed in a stereotaxic frame and the head was flexed ventrally. Neck muscles and the atlanto-occipital membrane were carefully bisected. 100 nl AAV was injected using a pulled glass micropipette A/P: 0.2 mm, D/V: −0.2 mm, M/L: 0 and +/- 0.2 mm relative to obex. Animals received oral carprofen analgesia on the day of surgery and 3 days post-operatively. Mice were allowed a 2 week recovery period prior to commencing studies.

AAVs were obtained from Addgene (AAV-*hSyn*-DIO-hM3D(Gq)-mCherry, 44361; AAV-*Ef1a*-mCherry-IRES-Cre, 55632).

Food Intake / body weight measurements in response to GLP-140/GIP-085 treatment

Gipr$^{\Delta\ Sst}$, *Gipr*$^{\Delta\ Hyp}$, and wild type litter mate control mice were challenged with an obesogenic diet (60% HFD, Research Diets) until they reached >40 g BW. Animals were then randomized to treatment groups based on body weight and acclimatised to single housing and

daily handling for 7 days. Following acclimatization, mice received daily subcutaneous injections of either vehicle, GLP-140 (30 nmol/kg), or GLP-140 (30 nmol/kg) + GIP-085 (300 nmol/kg) for 12 days. Daily body weight and food intake were measured throughout the study. Changes in body weight were calculated as a percentage of the body weight of the same animal prior to the first injection.

Calorimetry/ambulatory activity/ food/fluid intake studies

Animals were acclimated to indirect calorimetry cages fitted with a continuous monitoring system (Promethion, Sable Systems) housed in a temperature and humidity-controlled cabinet for 30 h prior to study and data collection. Oxygen consumption and carbon dioxide production were recorded and the respiratory exchange ratio (RER; VCO_2/VO_2) and energy expenditure (via the Weir equation, $EE = 3.941 \times VO_2 + 1.106 \times VCO_2$) were calculated. Ambulatory activity was measured via infrared beam breaks. Fluid and food consumption was recorded using hoppers connected to load cells for continuous monitoring. Animals were given standard chow and drinking water, drinking water with a choice of chow or 45% HFD, or a choice of drinking water or 10% sucrose with standard chow, as indicated. Raw data was processed using ExpeData (Sable Systems).

Conditioned taste avoidance (CTA) assay

Conditioned taste avoidance (CTA) assays were performed using a protocol modified from Chen *et al* (23). Mice were individually housed in cages fitted with two test tubes containing normal drinking water, and were allowed to acclimatize to the single housing, water bottles, and daily handling for 7 days. Following the acclimatization period, mice began the CTA protocol. On day 1 mice were fasted overnight for 12 h. Following the onset of the light phase on day 2, mice were re-fed and given 45 min access to a novel 5% sucrose solution, followed by injection of either CNO (IP, 1 mg/kg), 10 nmol/Kg GIP-532 (SC, 10 nmol/kg,(24)), LiCl (IP, 0.2 M), or vehicle control. Following conditioned stimulus pairing normal water was returned. Mice were fasted overnight for 12 h on day 3. Conditioning was repeated on day 4.

Mice were fasted overnight for 12 h on day 5. On day 6 mice were given one bottle containing normal drinking water and one bottle containing 5% sucrose. Placement of the water bottles was randomized across all animals. Bottle intake was recorded after four hours and the sucrose preference ratio was calculated as follows: (volume sucrose consumed)/ (total volume sucrose + water consumed).

C-Fos analysis

Mice were acclimatized to daily handling for 14 days before being dosed with either 1 mg/kg CNO or vehicle control (IP). 90 min post-injection, mice were terminally anaesthetised with sodium pentobarbital (200 mg/kg) and transcardially perfused with 0.1M PBS followed by 4% PFA. Prior to sacrifice mice had access to food and water. Brains were harvested and processed for IHC using c-Fos antisera as described above. C-Fos positive nuclei were quantified manually using Halo image analysis software (Indica Labs).

Data availability

scRNAseq data are available from the NCBI Gene Expression Omnibus accession GSE228192.

Statistics

Data are presented as mean and SEM. Statistical analysis was performed using Microsoft Excel, GraphPad Prism 7.0, and Seurat V4. For all statistical tests, an α risk of 5% was used. Multiple comparisons were made using a 2-way ANOVA or a repeated measures 2-way ANOVA with a post-hoc Tukey or Sidak test, as indicated in the figure legends. Single comparisons were made using either a paired or unpaired Student's t tests where appropriate as indicated in the figure legends. Sample size was computed based on pilot data and previously published data. N numbers represent the number of mice or biological replicates used in each study unless otherwise indicated in the figure legend.

Supplementary Methods and Materials References

1. Adriaenssens AE, Biggs EK, Darwish T, Tadross J, Sukthankar T, Girish M, et al. Glucose-Dependent Insulinotropic Polypeptide Receptor-Expressing Cells in the Hypothalamus Regulate Food Intake. *Cell Metab.* 2019;30(5):987-96.e6.
2. Richards P, Parker HE, Adriaenssens AE, Hodgson JM, Cork SC, Trapp S, et al. Identification and characterization of GLP-1 receptor-expressing cells using a new transgenic mouse model. *Diabetes.* 2014;63(4):1224-33.
3. Zariwala HA, Borghuis BG, Hoogland TM, Madisen L, Tian L, De Zeeuw CI, et al. A Cre-dependent GCaMP3 reporter mouse for neuronal imaging in vivo. *J Neurosci.* 2012;32(9):3131-41.
4. Joo E, Harada N, Yamane S, Fukushima T, Taura D, Iwasaki K, et al. Inhibition of Gastric Inhibitory Polypeptide Receptor Signaling in Adipose Tissue Reduces Insulin Resistance and Hepatic Steatosis in High-Fat Diet-Fed Mice. *Diabetes.* 2017;66(4):868-79.
5. Adriaenssens A, Lam BY, Billing L, Skeffington K, Sewing S, Reimann F, et al. A Transcriptome-Led Exploration of Molecular Mechanisms Regulating Somatostatin-Producing D-Cells in the Gastric Epithelium. *Endocrinology.* 2015;156(11):3924-36.
6. Campbell JE, Ussher JR, Mulvihill EE, Kolic J, Baggio LL, Cao X, et al. TCF1 links GIPR signaling to the control of beta cell function and survival. *Nat Med.* 2016;22(1):84-90.
7. Borner T, Geisler CE, Fortin SM, Cosgrove R, Alsina-Fernandez J, Dogra M, et al. GIP Receptor Agonism Attenuates GLP-1 Receptor Agonist-Induced Nausea and Emesis in Preclinical Models. *Diabetes.* 2021;70(11):2545-53.
8. Samms RJ, Christe ME, Collins KA, Pirro V, Droz BA, Holland AK, et al. GIPR agonism mediates weight-independent insulin sensitization by tirzepatide in obese mice. *J Clin Invest.* 2021;131(12).
9. Perens J, Salinas CG, Skytte JL, Roostalu U, Dahl AB, Dyrby TB, et al. An Optimized Mouse Brain Atlas for Automated Mapping and Quantification of Neuronal Activity Using iDISCO+ and Light Sheet Fluorescence Microscopy. *Neuroinformatics.* 2021;19(3):433-46.
10. Ludwig MQ, Cheng W, Gordian D, Lee J, Paulsen SJ, Hansen SN, et al. A genetic map of the mouse dorsal vagal complex and its role in obesity. *Nat Metab.* 2021;3(4):530-45.
11. Zhang C, Kaye JA, Cai Z, Wang Y, Prescott SL, and Liberles SD. Area Postrema Cell Types that Mediate Nausea-Associated Behaviors. *Neuron.* 2021;109(3):461-72.e5.
12. Smith C, Patterson-Cross R, Woodward O, Lewis J, Chiarugi D, Merkle F, et al. A comparative transcriptomic analysis of glucagon-like peptide-1 receptor- and glucose-dependent insulinotropic polypeptide-expressing cells in the hypothalamus. *Appetite.* 2022;174:106022.
13. Hao Y, Hao S, Andersen-Nissen E, Mauck WM, Zheng S, Butler A, et al. Integrated analysis of multimodal single-cell data. *Cell.* 2021;184(13):3573-87.e29.
14. Marques S, Zeisel A, Codeluppi S, van Bruggen D, Mendanha Falcão A, Xiao L, et al. Oligodendrocyte heterogeneity in the mouse juvenile and adult central nervous system. *Science.* 2016;352(6291):1326-9.
15. Zeisel A, Hochgerner H, Lönnerberg P, Johnsson A, Memic F, van der Zwan J, et al. Molecular Architecture of the Mouse Nervous System. *Cell.* 2018;174(4):999-1014.e22.
16. Campbell JN, Macosko EZ, Fenselau H, Pers TH, Lyubetskaya A, Tenen D, et al. A molecular census of arcuate hypothalamus and median eminence cell types. *Nat Neurosci.* 2017;20(3):484-96.
17. Lein ES, Hawrylycz MJ, Ao N, Ayres M, Bensinger A, Bernard A, et al. Genome-wide atlas of gene expression in the adult mouse brain. *Nature.* 2007;445(7124):168-76.
18. Chen R, Wu X, Jiang L, and Zhang Y. Single-Cell RNA-Seq Reveals Hypothalamic Cell Diversity. *Cell Rep.* 2017;18(13):3227-41.
19. Mickelsen LE, Flynn WF, Springer K, Wilson L, Beltrami EJ, Bolisetty M, et al. Cellular taxonomy and spatial organization of the murine ventral posterior hypothalamus. *Elife.* 2020;9.

20. Mickelsen LE, Bolisetty M, Chimileski BR, Fujita A, Beltrami EJ, Costanzo JT, et al. Single-cell transcriptomic analysis of the lateral hypothalamic area reveals molecularly distinct populations of inhibitory and excitatory neurons. *Nat Neurosci.* 2019;22(4):642-56.
21. Moffitt JR, Bambah-Mukku D, Eichhorn SW, Vaughn E, Shekhar K, Perez JD, et al. Molecular, spatial, and functional single-cell profiling of the hypothalamic preoptic region. *Science.* 2018;362(6416).
22. He L, Vanlandewijck M, Raschperger E, Andaloussi Mäe M, Jung B, Lebouvier T, et al. Analysis of the brain mural cell transcriptome. *Sci Rep.* 2016;6:35108.
23. Chen JY, Campos CA, Jarvie BC, and Palmiter RD. Parabrachial CGRP Neurons Establish and Sustain Aversive Taste Memories. *Neuron.* 2018;100(4):891-9.e5.
24. Samms RJ, Cosgrove R, Snider BM, Furber EC, Droz BA, Briere DA, et al. GIPR Agonism Inhibits PYY-Induced Nausea-Like Behavior. *Diabetes.* 2022;71(7):1410-23.
